# MitoPerturb-Seq identifies gene-specific single-cell responses to mitochondrial DNA depletion and heteroplasmy

**DOI:** 10.1038/s41594-026-01779-7

**Published:** 2026-04-01

**Authors:** Stephen P. Burr, Kathryn Auckland, Angelos Glynos, Abhilesh Dhawanjewar, Cameron Ryall, Wei Wei, Antony Hynes-Allen, Malwina Prater, Matylda Sczaniecka-Clift, Julien Prudent, Patrick F. Chinnery, Jelle van den Ameele

**Affiliations:** 1 MRC Mitochondrial Biology Unit, University of Cambridge, Cambridge, UK; 2 Department of Clinical Neurosciences, University of Cambridge, Cambridge, UK; 3 MRC Laboratory of Molecular Biology, University of Cambridge, Cambridge, UK; 4 Present Address: Altos Labs Cambridge Institute, Cambridge, UK

**Keywords:** High-throughput screening, Genomics, Mitochondria

## Abstract

Mitochondria contain their own genome, mitochondrial DNA (mtDNA), which is under strict control by the cell nucleus. mtDNA occurs in many copies per cell and mutations often only affect a proportion of them, giving rise to heteroplasmy. mtDNA copy number and heteroplasmy level together shape the tissue-specific impact of mtDNA mutations, eventually giving rise to both rare mitochondrial and common neurodegenerative diseases. Here, we use MitoPerturb-Seq for CRISPR–Cas9-based, high-throughput single-cell interrogation of the nuclear genes and pathways that sense and control mtDNA copy number and heteroplasmy. We screened a panel of mtDNA maintenance genes in mouse cells with a heteroplasmic mtDNA *mt-Ta* mutation. This revealed both common and perturbation-specific aspects of the integrated stress response to mtDNA depletion caused by *Tfam*, *Opa1* and *Polg* knockout. These responses are only partially mediated by ATF4 and cause cell-cycle stage-independent slowing of cell proliferation. MitoPerturb-Seq, thus, provides experimental insight into disease-relevant mitochondrial–nuclear interactions and may inform development of therapies targeting cell-type- and tissue-specific vulnerabilities to mitochondrial dysfunction.

## Abstract (web summary)

Burr and Auckland et. al develop MitoPerturb-Seq, which combines single-cell screening with multiomics to link nuclear genes to mitochondrial DNA (mtDNA) dynamics. They unveil core regulators of mtDNA copy number and characterize cell-cycle delays and transcriptional stress in response to mtDNA depletion.

## Main

Mitochondria store their own genetic information in a ~16.5-kb small circular genome, the mtDNA, which encodes 37 genes required for mitochondrial oxidative phosphorylation (OXPHOS). Each cell may contain hundreds to thousands of copies of mtDNA and their replication and transcription are strictly controlled by nuclear-encoded mitochondrial localized proteins. mtDNA copy number (CN), the absolute number of mtDNA molecules per cell, shows considerable tissue-specific and cell-type-specific variability^1,2^. Furthermore, an age-dependent decrease in mtDNA CN may contribute to aging and neurodegeneration^2,3^. Mutations in the mtDNA often only affect a proportion of mtDNA molecules within a cell, a state called heteroplasmy^4,5^. During development and aging, the number and proportion of mutated mtDNA molecules can increase to high levels in single cells. Symptoms arise when deleterious heteroplasmy levels reach a cell-type-specific threshold, leading to biochemical OXPHOS defects, cell dysfunction and cell death. Age-related clonal expansion of mtDNA mutations has been implicated in the pathogenesis of rare mitochondrial diseases^6^ and common neurodegenerative disorders, including Alzheimer’s and Parkinson’s disease^7,8^, as well as in specific types of cancer^9^. Identification of factors and pathways that regulate mtDNA CN and reduce heteroplasmy, even by a few percent below the threshold, offers the possibility to reverse biochemical defects and confer protection against a range of rare and common diseases.

Genome-wide association studies have shown the importance of nuclear loci modulating mtDNA CN and heteroplasmy but little is known about the specific genes involved. Most studies to date have been correlative and were based on bulk tissue analysis containing diverse cell types in varying proportions, with inconsistent and contradictory findings^4,10–16^. As a result, for many nuclear-encoded genes and genomic loci, a direct causal link between gene activity and mtDNA dynamics remains to be established. Moreover, given the extraordinary degree of mtDNA mosaicism across tissues, with virtually all cells containing different mixtures and levels of wild-type (WT) and mutant mtDNA^17–19^, it remains unclear how mtDNA CN and heteroplasmy levels interact within individual cells to cause a downstream tissue-specific phenotype.

Here, we describe and deploy MitoPerturb-Seq, allowing us to determine the impact of specific nuclear genetic perturbations on mtDNA CN, heteroplasmy and the resulting transcriptomic response at single-cell resolution. Targeting a library of candidate nuclear genetic modifiers of mtDNA dynamics in heteroplasmic mouse embryonic fibroblasts (MEFs), we show gene-specific modulation of mtDNA CN and heteroplasmy variance, affecting cell-cycle progression and the associated nuclear transcriptional response. DamID-seq-based chromatin profiling confirmed ATF4 as a key mediating transcription factor for some but not all nuclear genes responding to decreased mtDNA levels. MitoPerturb-Seq, thus, offers a unique opportunity for unbiased high-throughput interrogation of disease-relevant interactions between nuclear and mitochondrial genomes within single, isogenic cells.

## Results

### Single-cell CRISPR screening with whole-cell multiome in heteroplasmic cells

**Fig. 1:**
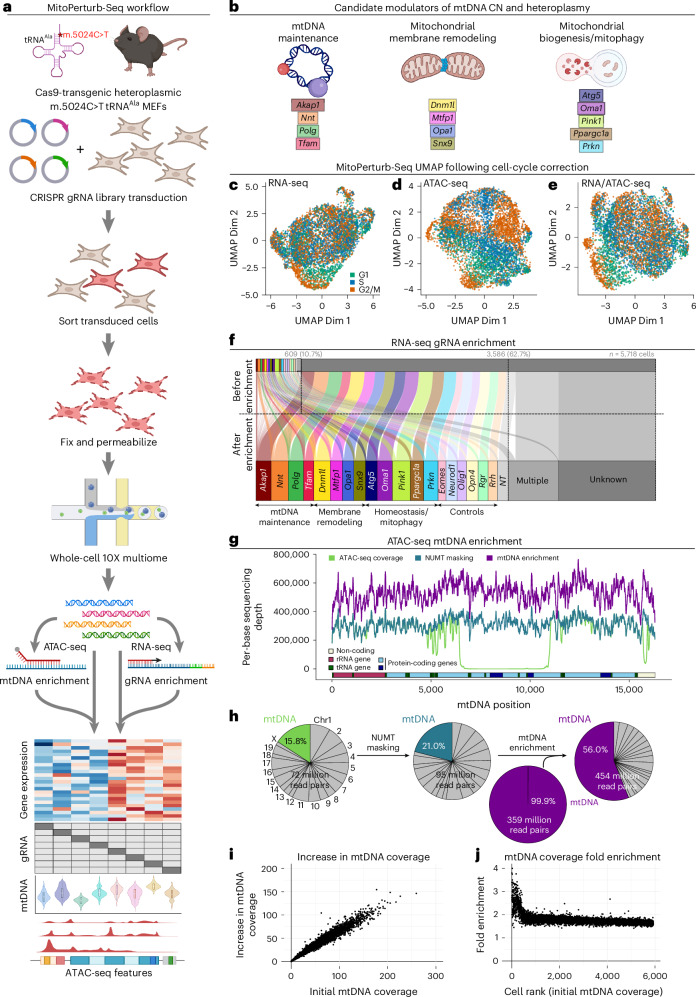
MitoPerturb-Seq combines single-cell CRISPR screening with whole-cell multiome in heteroplasmic cells. **a**, Overview of the MitoPerturb-Seq experimental workflow in heteroplasmic MEFs. **b**, Candidate genes included in the MitoPerturb-Seq pooled gRNA library. **c**–**e**, UMAPs showing MitoPerturb-Seq cells, clustered on the basis of overall RNA-seq expression (**c**), chromatin accessibility (**d**) and a combined WNN analysis (**e**), following regression of cell-cycle heterogeneity from the RNA-seq dataset. Cells are colored by cell-cycle phase. **f**, MitoPerturb-Seq target gene assignments before and after gRNA enrichment. Target genes are colored as in **b**. The total number of cells and the number of cells with gRNA assignments before and after enrichment are indicated above the plot. **g**,**h**, Per-base coverage (**g**) or percentage of ATAC-seq reads (**h**) aligning to the mtDNA, following alignment to the standard mm10 genome (light green) or to an NUMT-masked mm10 genome before (dark green) and after (purple) hybridization-capture-based mtDNA enrichment. **i**,**j**, Per-cell absolute (**i**) or fold (**j**) increase in mtDNA coverage following mtDNA enrichment, compared to initial coverage before enrichment (**i**) or to cells ordered by initial mtDNA coverage (**j**). Figure created in BioRender. Van den Ameele, J. (2026) https://BioRender.com/jpd1min.

**Extended Data Fig. 1:**
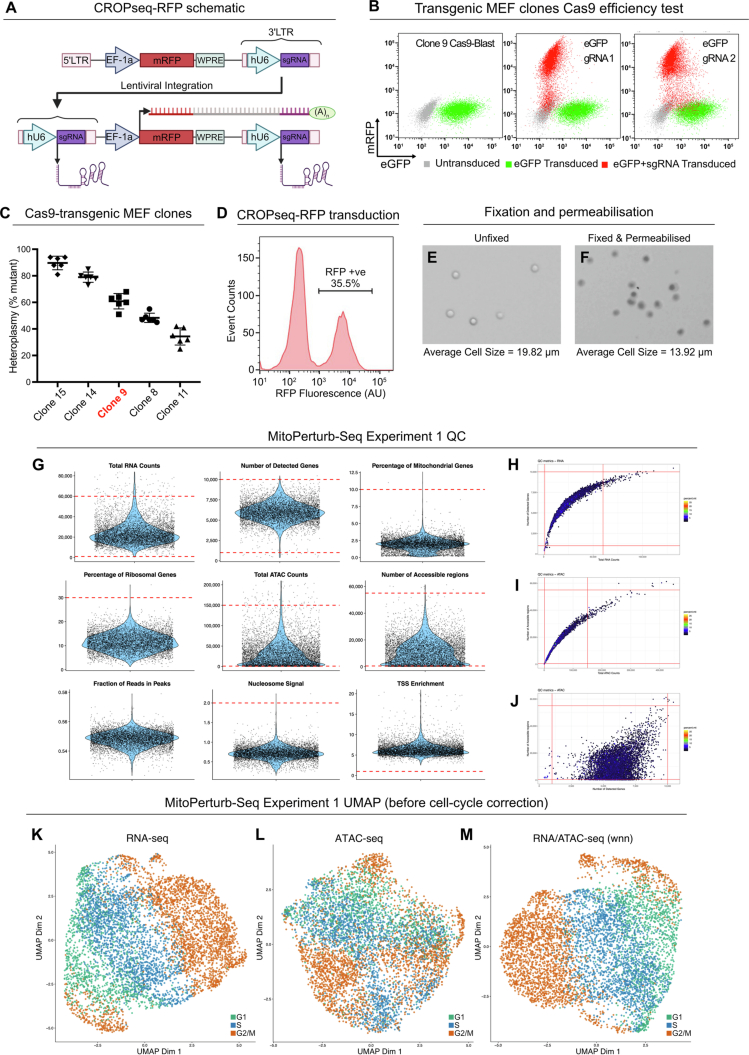
MitoPerturb-Seq and quality-control steps. (**A**) Schematic of the CROPseq-RFP lentiviral cassette showing transcript expression and poly-adenylation of a gRNA-containing transcript following genomic integration. (**B**) Flow cytometry plots confirming genome editing efficiency in transgenic Cas9-expressing MEF clones. Clonal cells stably expressing eGFP were transduced with CROPseq-RFP lentivirus carrying two separate gRNAs targeting the eGFP sequence. RFP-high cells displayed loss of eGFP fluorescence, confirming effective CRISPR-Cas9 editing of the target gene. (**C**) Single-cell heteroplasmy measurements from five Cas9 transgenic MEF clones, six cells per clone. Clone 9, used for MitoPerturb-Seq experiments, is highlighted in red. Data presented as mean values ± SD. (**D**) Flow cytometry plot showing RFP expression 10 days post-transduction with the pooled CROPseq-RFP gRNA library. Transduction efficiency of 35.5% (MOI = 0.35) indicates that the majority of cells received a single viral particle. (**E,F**) Bright field images of unfixed/unpermeabilized (**E**) or fixed/permeabilized (**F**) MEFs stained with trypan blue. Viable cells (**E**) have not taken up the dye and remain bright with a well-defined membrane, while permeabilized cells (**F**) have taken up the dye, with darker-staining nuclei. Average cell size, calculated by the Countess cell counter, is indicated. (**G**) QC violins from Seurat for RNA-seq and ATAC-seq, dashed red lines indicate thresholds used for filtering, as described in the methods. Outliers with high y-axis values have been removed from selected plots: Total RNA Counts, 27 outliers; Percentage of Mitochondrial Genes, 2 outliers; Total ATAC Counts, 47 outliers; TSS Enrichment 6 outliers. (**H-J**) QC plots of nCountRNA (**H**), nCountATAC (**I**) and nFeatureRNA (**J**) versus nFeatureRNA (**H**) or nFeatureATAC (**I,J**), colored by percent mitochondrial reads, with lines indicating cutoff thresholds shown in violin plots in (**G**). (**K-M**) UMAPs showing MitoPerturb-Seq cells, clustered based on overall RNA-seq (**K**), ATAC-seq (**L**) or by Weighted Nearest Neighbor (WNN) of combined RNA- and ATAC-seq (**M**) data, prior to regression of cell cycle heterogeneity. Each point represents an individual cell. Cells are colored by cell cycle phase. Figure created in BioRender. Van den Ameele, J. (2026) https://BioRender.com/j3ro7vq. Source data

**Extended Data Fig. 2:**
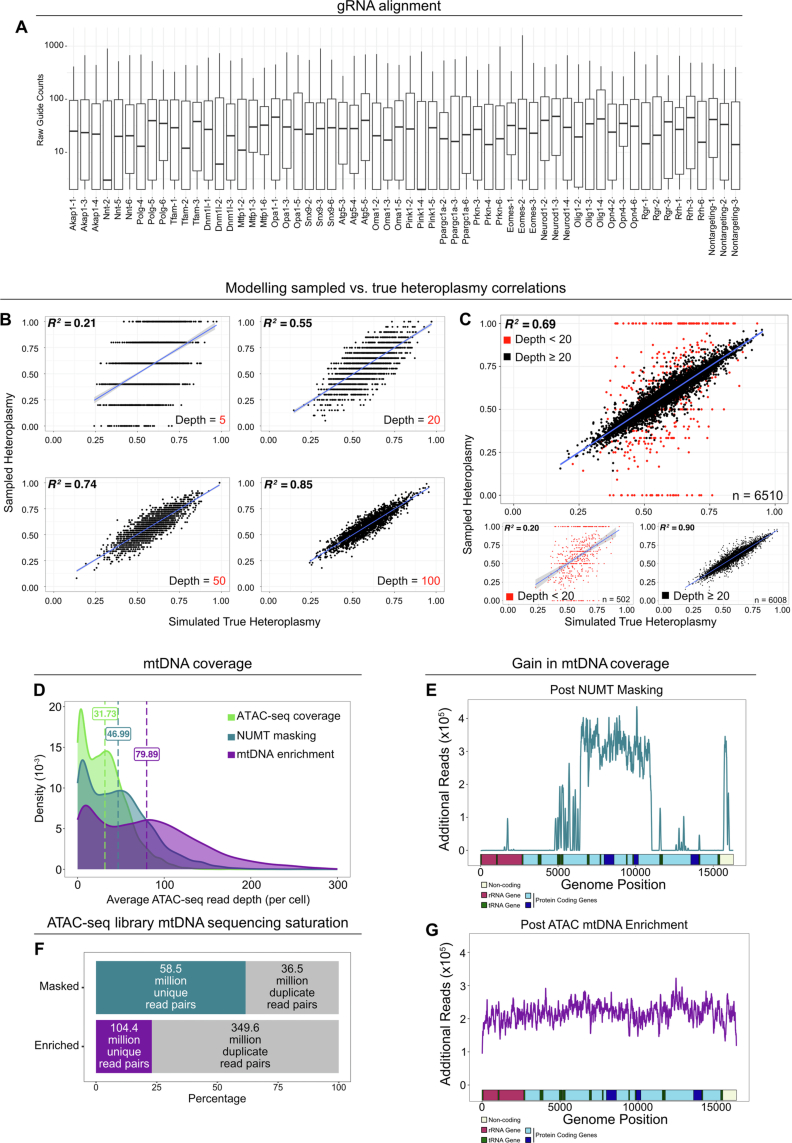
gRNA detection and targeted enrichment of mtDNA. (**A**) Raw read counts per cell aligned to each of the gRNA sequences included in the gRNA library. For each gRNA, all cells with at least 2 counts are shown, cell numbers per gRNA group are provided in Supplementary Table 3. Boxes encompass 25^th^-75^th^ percentiles, mid-line indicates median, whiskers extend to extreme values, no data points fall outside of the whisker range. (**B**) Sampled heteroplasmy vs. true heteroplasmy correlation at different mtDNA coverage. A population of 2,000 cells was simulated, and randomly sub-sampled at increasing sequencing depth, with sampled heteroplasmy calls showing better correlation with true heteroplasmy as sampling depth increases. Fit line displays smoothed conditional mean, shaded regions around the fit line are 95% CI. (**C**) Sampled heteroplasmy vs. true heteroplasmy in an *in silico* simulated version of the MitoPerturb-Seq dataset. A population of 6,510 cells was simulated, and sub-sampled using the same mtDNA coverage distribution as obtained from post-mtDNA enrichment mgatk analysis. Cells sampled at depth <20 (n = 502) are highlighted in red, confirming that a depth threshold of ≥ 20 successfully removed cells with the most inaccurate heteroplasmy calls. Smaller panels plot the <20 depth and ≥ 20 depth populations from the same simulation separately, with corresponding R^2^ values and cell numbers shown. Fit line displays smoothed conditional mean, shaded regions around the fit line are 95% CI. (**D**) Distribution of mtDNA coverage in the MitoPerturb-Seq dataset following alignment of ATAC-seq reads to the standard mm10 reference (light green) and to a NUMT-masked version of mm10 pre- (dark green) and post- (purple) mtDNA hybridization capture-based enrichment. Mean mtDNA coverage values for each approach are indicated. (**E**) Line plot showing per-base additional mtDNA coverage gained when aligning ATAC-seq reads to a NUMT-masked version of the mm10 genome compared to the standard mm10 reference. (**F**) Percentage of unique vs duplicate sequencing reads in pre- and post-mtDNA enrichment ATAC-seq libraries. Raw read-pair numbers for each library are indicated. (**G**) Line plot showing per-base additional mtDNA coverage gained following hybridization capture enrichment of mtDNA-specific sequences, aligned to the NUMT-masked mm10 genome.

We established MitoPerturb-Seq to understand whether disruption of candidate nuclear genes modulates mtDNA at the single-cell level. This would allow us to identify genes involved in regulating mutation burden and/or mtDNA CN in cells with heteroplasmic mtDNA mutations, where changes in these parameters are known to affect disease phenotypes^20,21^. MitoPerturb-Seq combines CROPseq for pooled single-cell CRISPR screening^22^ with a 10X Genomics multiome-based approach^23,24^ for combined single-cell (sc)ATAC-seq and scRNA-seq in whole cells. This enables simultaneous profiling of mtDNA sequence, CN and heteroplasmy from scATAC-seq and the detection of guide RNA (gRNA) sequences from the single-cell transcriptome (Fig. 1a and Extended Data Fig. 1a). We generated Cas9-expressing MEFs (Extended Data Fig. 1b) from heteroplasmic mice carrying an m.5024C>T point mutation in the mitochondrial tRNA^Ala^ gene (*mt-Ta*)^11,25^, which corresponds to the human disease-causing heteroplasmic m.5650G>A tRNA^Ala^ variant^25–27^ (Fig. 1a). Selected Cas9-transgenic MEFs had a mean heteroplasmy of 60.8% ± 5.8% (mean ± s.d.), just below the threshold for biochemical defects in most tissues^25^, with low intercellular variability (Extended Data Fig. 1c). MEFs were transduced with a pooled gRNA library (60 gRNAs, three gRNAs per gene, six control genes, three nontargeting (NT) gRNAs) to perturb 13 nuclear genes encoding proteins previously suggested to affect mtDNA CN and/or heteroplasmy, either through their role in mtDNA maintenance (*Akap1* (ref.^28^), *Nnt*^13^, *Polg*^12,29^ and *Tfam*^10,30,31^), mitochondrial membrane remodeling (*Dnm1l* (also known as *Drp1*)^32^, *Mtfp1* (ref. ^33^), *Opa1* (ref. ^34,35^) and *Snx9* (ref. ^36,37^) or mitochondrial biogenesis and mitophagy (*Atg5* (ref. ^38^), *Oma1* (ref. ^13^), *Pink1* (ref. ^28,39^), *Ppargc1a*^40^ and *Prkn*^39,41^) (Fig. 1b and Supplementary Table 1). Then, 10 days after transduction, gRNA-expressing cells were processed for whole-cell multiome (Fig. 1a and Extended Data Fig. 1d–f). Following quality control (QC) of raw sequencing reads, barcode processing and cell filtering, 5,718 high-confidence single cells were identified, with an average of 5,967 genes and 11,783 unique ATAC-seq peaks per cell; data were visualized by weighted nearest neighbor (WNN) uniform manifold approximation and projection (UMAP) after cell-cycle correction (Fig. 1c–e and Extended Data Fig. 1g–m). In total, 0.0022% of RNA-seq reads aligned to gRNAs (Extended Data Fig. 2a) and 15.8% of ATAC-seq reads aligned to mtDNA, corresponding to 31.7× mean mtDNA sequencing depth per cell.

**Extended Data Fig. 3:**
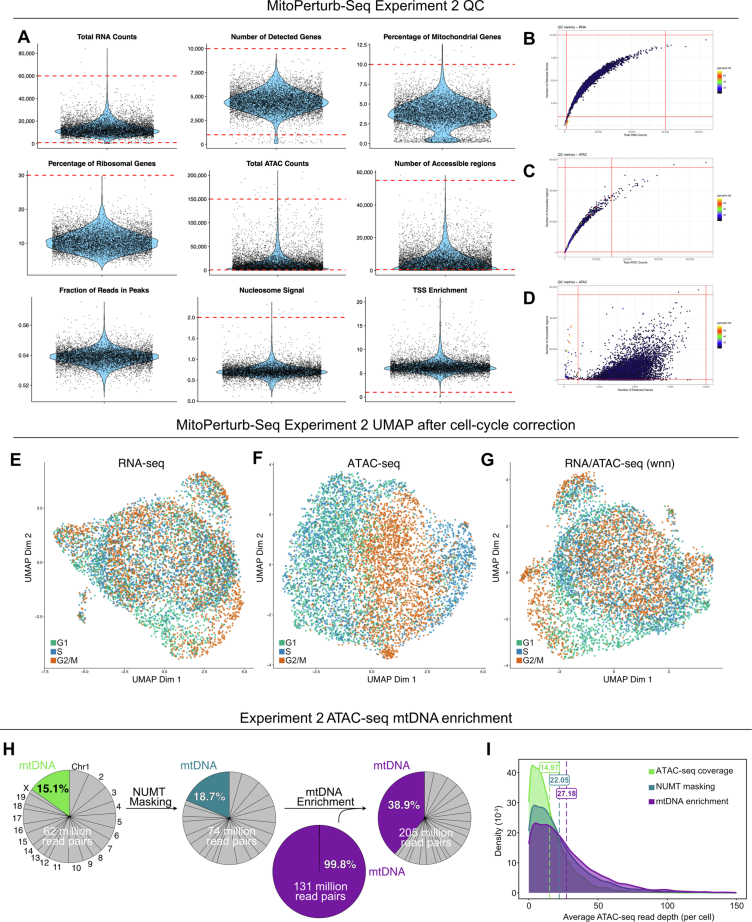
Quality control steps of experiment 2. (**A**) QC violins from Seurat for RNA-seq and ATAC-seq, dashed red lines indicate thresholds used for filtering, as described in the methods. Outliers with high y-axis values have been removed from selected plots: Percentage of Mitochondrial Genes, 27 outliers; Total ATAC Counts, 16 outliers; TSS Enrichment, 30 outliers. (**B-D**) QC plots of nCountRNA (**B**), nCountATAC (**C**) and nFeatureRNA (**D**) versus nFeatureRNA (**B**) or nFeatureATAC (**C,D**), colored by percent mitochondrial reads, with lines indicating cutoff thresholds shown in violin plots in (**A**). (**E-G**) UMAPs showing cells from the MitoPerturb-Seq dataset in Experiment 2, clustered based on overall RNA expression patterns (**E**), chromatin accessibility profiles (**F**) and a combined Weighted Nearest Neighbor (WNN) analysis (**G**), following regression of cell cycle heterogeneity from the RNA-seq dataset. Each point represents an individual cell. Cells are colored by cell cycle phase. (**H,I**) Percentage of ATAC-seq reads (**H**) and distribution of mtDNA coverage (**I**) in the replicate MitoPerturb-Seq dataset aligning to the mtDNA using the standard mm10 reference (light green), a NUMT-masked version of the mm10 genome (dark green), and following addition of enriched mtDNA reads following hybridization capture (purple). Mean coverage values for each group are indicated in (**I**).

**Extended Data Fig. 4:**
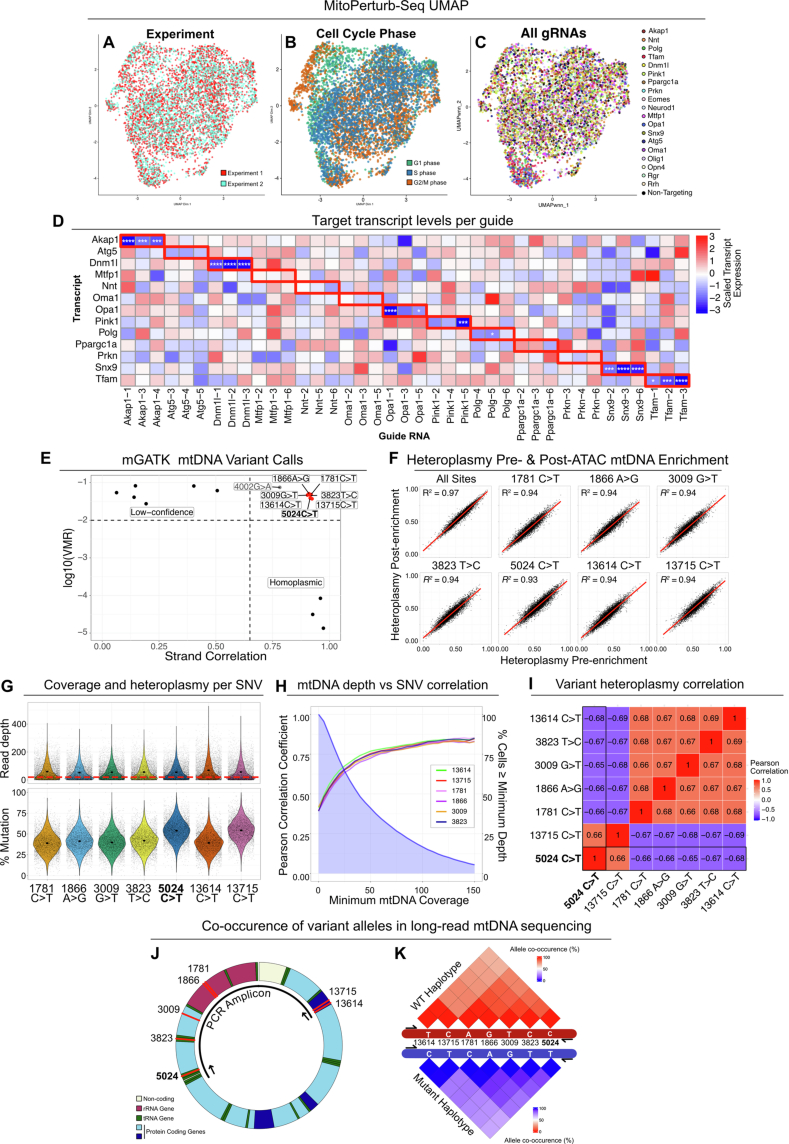
Data-integration, gRNA efficiency, co-segregation of heteroplasmic mtDNA variants. (**A-C**) UMAPs of the integrated MitoPerturb-Seq dataset, WNN clustered following cell cycle regression from RNA-seq. Cells are colored by technical replicate (**A**), cell cycle phase (**B**) and target gene gRNA assignment (**C**). (**D**) Scaled transcript levels for each target gene across all perturbation groups, split by individual gRNA assignment. Expression values normalized per-column. Two-tailed t-tests comparing target transcript expression in the relevant perturbation group vs NT gRNA group, * p < 0.05, *** p < 0.001, **** p < 0.0001. (**E**) High-confidence heteroplasmic variants present in the m.5024 C > T MEFs, identified by mgatk. Seven variants (red) were previously reported, with an additional variant, m.4002 G > A, likely to be a clone-specific *de novo* mutation not present in the mouse strain. (**F**) Correlation of per-cell heteroplasmy calls pre- and post-mtDNA enrichment from the scATACseq library at read-depth ≥20 for each individual SNV and all 7 sites combined. (**G**) Per-cell read depth (top) and heteroplasmy (bottom) at each of the seven main variant sites (SNVs). Heteroplasmy distributions match the reported linkage of m.5024 C > T *in cis* with m.13715 C > T and *in trans* with m.1781 C > T, m.1866A > G, m.3009 G > T, m.3823 T > C and m.13614 C > T^58^. On the heteroplasmy plot, only cells with mtDNA coverage ≥20 (indicated by the red line on the upper panel) at the corresponding SNV position are shown. (**H**) Pearson correlation coefficients (left axis) between heteroplasmy calls at each SNV and m.5024 C > T with increasing minimum read depth threshold (horizontal axis). Blue shaded area indicates the percentage of cells above the depth threshold (right axis). (**I**) Pearson correlation between all variant sites with minimum mtDNA depth threshold ≥20. (**J,K**) Schematic of the mtDNA showing the location of the seven main SNVs and the PCR amplicon (**J**) used for long-read sequencing of mtDNA from skin biopsies of 2-week-old m.5024 C > T mice (1 male & 2 female) (**K**). Percentage of long-reads with co-occurrence of each allele with any of the other alleles on the same haplotype (WT, red, top; mutant, blue, bottom). Co-occurrence is less strong for distant SNVs, due to PCR-mediated recombination. SNV positions are highlighted in red. Source data

To improve gRNA assignment, we used TAP-seq^42^ for PCR-based enrichment of gRNA transcripts. This increased the number of cells assigned to a specific gRNA from 609 (10.7%) to 3,586 (62.7%), expanding the number of cells in each target gene group to 179 ± 41 (mean ± s.d.) (Fig. 1f). As the confidence of estimating true heteroplasmy levels increases with higher read depth^43^ (Extended Data Fig. 2b,c), we also sought to further increase single-cell mtDNA coverage. Masking nuclear-localized mitochondrial sequences (NUMTs) in the reference genome to prevent dual alignment to mtDNA and NUMT sequences^17^ increased average sequencing depth per cell to 47× ± 37× (21.0% of ATAC-seq reads) (Fig. 1g,h and Extended Data Fig. 2d,e). In addition, we used a hybridization-capture-based enrichment of mtDNA from scATAC-seq libraries (Fig. 1a and Supplementary Table 2), similar to the recently reported ReDeeM approach^44^, to generate a separate sequencing library consisting of 99.9% mtDNA reads (Fig. 1h), with higher sequencing saturation (mtDNA read duplication rate increased from 38% to 77%) (Extended Data Fig. 2f), evenly spread across the mitochondrial genome (Fig. 1g and Extended Data Fig. 2g). This increased the average mtDNA sequencing depth per cell ~1.7-fold, regardless of initial mtDNA coverage, except in those cells with very low initial coverage (<2×), where depth rose 2–3-fold (Fig. 1i,j). Together, this resulted in an 80× ± 61× average mtDNA sequencing depth per cell (Extended Data Fig. 2d). A replicate experiment under identical conditions resulted in 6,272 cells (Extended Data Fig. 3a–g), of which 2,965 (43%) could be assigned a unique gRNA identity, with 31.7× ± 30× mtDNA coverage after enrichment (Extended Data Fig. 3h,i). A range of QC metrics showed consistent results across both replicates (Extended Data Figs. 1g–j and 3a–d) and UMAP clustering of both datasets displayed an even distribution of cells across all clusters (Extended Data Fig. 4a), confirming the reproducibility of our MitoPerturb-Seq approach in heteroplasmic MEFs.

### MitoPerturb-Seq identifies mtDNA depletion following targeted gene perturbation

**Fig. 2:**
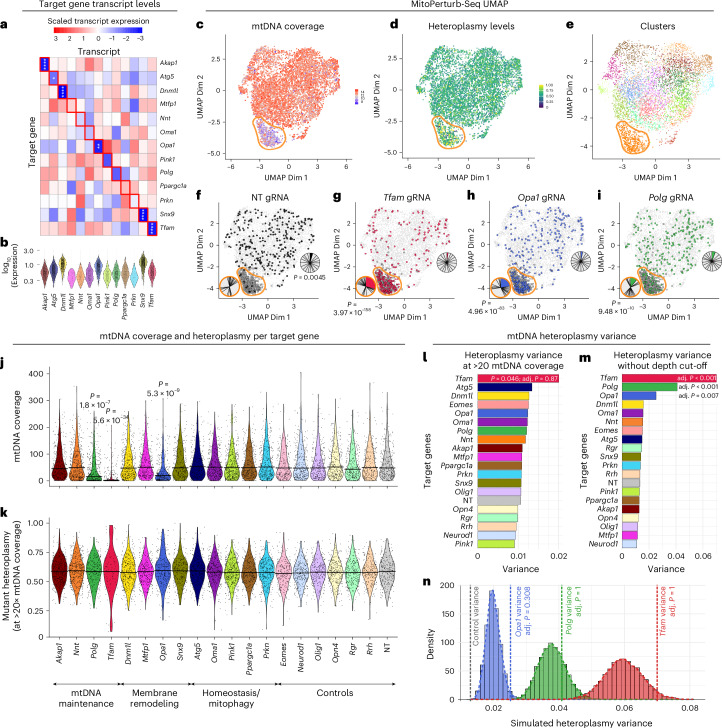
MitoPerturb-Seq identifies mtDNA depletion following targeted gene perturbation. **a**,**b**, Scaled transcript levels (**a**) and per-cell log-transformed transcript counts (**b**) of candidate target genes across all perturbation groups. Expression values normalized on a per-column basis in **a**; cells with zero counts are not plotted in **b**. Two-tailed *t*-tests were used to evaluate statistical differences in target transcript expression in the relevant perturbation group versus NT gRNA group. *P < 0.05, **P < 0.01 and ****P < 0.0001. **c**–**e**, UMAP shaded according to mtDNA coverage (**c**) or heteroplasmy level (**d**) and following guided clustering (**e**). Cluster 4 is highlighted by the yellow outline. **f**–**i**, UMAP with cells assigned to NT (**f**; black), *Tfam* (**g**; red), *Opa1* (**h**; blue) and *Polg* (**i**; green) gRNAs highlighted. Pie charts indicate the distribution of perturbation groups in cluster 4 (indicated by yellow outline and dark gray points) or all other clusters. Chi-squared tests were used to evaluate statistical differences. Adjusted *P* values for all perturbation groups are shown in Extended Data Fig. 5b. **j**,**k**, Per-cell mtDNA coverage (**j**) and heteroplasmy calls after read-depth filtering (**k**), for each perturbation group including controls. Each violin represents the combined data from all three gRNAs targeting the corresponding gene. Only cells with a combined read depth of ≥20 at the seven heteroplasmic SNVs are shown in **k**. Pairwise *t*-tests with multiple testing correction (Bonferroni) were used to evaluate statistical differences. *P* values are specified in the figure, with all *P* values < 0.05 shown. Horizontal lines indicate the median (**j**) or mean (**k**). **l**,**m**, Variance in heteroplasmy levels for each perturbation group, using only cells with >20 mtDNA read depth (**l**) or without read-depth filtering (**m**). Statistical significance was assessed using Brown-Forsythe tests (one-way analysis of variance (ANOVA)) against NT control group, with Bonferroni–Holm multiple testing correction. **n**, Heteroplasmy variance distributions from simulations modeling the stochastic sampling bottleneck. Control group heteroplasmies were downsampled to the read depths of *Opa1*-KD, *Polg*-KD and *Tfam*-KD groups (*n* = 5,000 simulations). Dashed lines indicate observed variances (colored) and the control group baseline (gray). Empirical two-tailed *P* values (Bonferroni–Holm-corrected) were determined.

We combined both experiments into a single integrated dataset (Extended Data Fig. 4a,b) to maximize the potential for discovery of perturbation-related mitochondrial phenotypes. This yielded 11,990 cells with 60.6× average mtDNA sequencing depth. The data were filtered to only retain cells with a unique gRNA assignment, resulting in a total of 6,551 cells (Extended Data Fig. 4c and Supplementary Table 3). As expected^45^, target gene knockdown (KD) efficiency for each gRNA was proportional to baseline expression level (Fig. 2a,b and Extended Data Fig. 4d). For example, Mtfp1 expression could only be detected at low levels (≤2 unique counts) in 4.6% of cells, likely explaining why average mRNA expression did not decrease with any of the three gRNAs targeting this gene.

**Extended Data Fig. 5:**
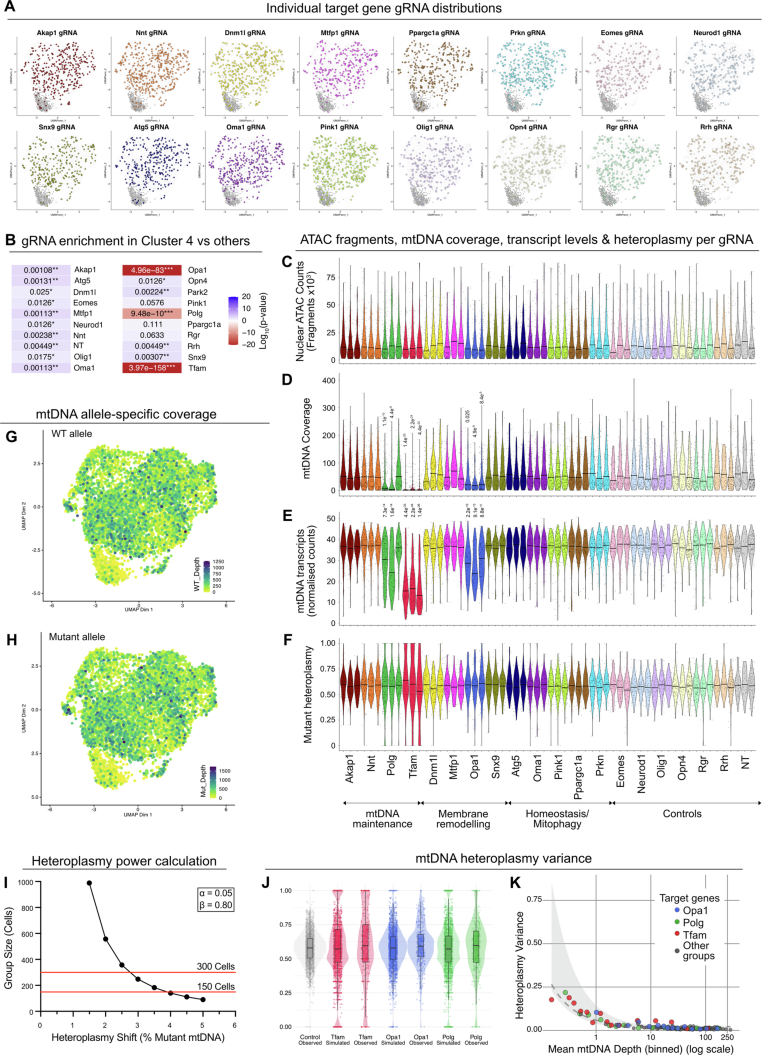
MitoPerturb-seq identifies mtDNA depletion following targeted gene perturbation. (**A**) Cells assigned to individual gRNA groups (NT, *Tfam*, *Opa1* and *Polg* UMAPs shown in Fig. 2f-i). (**B**) Relative enrichment of individual gRNA groups in cluster 4 (low mtDNA CN) compared to all other clusters. Target genes highlighted red are over-represented and blue under-represented in cluster 4. Two-sided chi-squared tests with multiple testing correction (Benjamini-Hochberg) and odds ratio calculations. All adjusted p-values shown; *p < 0.05, **p < 0.01, ***p < 0.001. NB: Scaled colouring is capped at p = 1×10^*−*20^ for clarity, exact p-values are on plots. (**C-F**) Per-cell nuclear ATAC fragment count (**C**) mtDNA coverage (**D**), mtDNA transcript levels (**E**) and heteroplasmy (**F**) for each gRNA group, including controls. Each violin represents one of three gRNAs targeting the corresponding gene. Pairwise two-sided Welch’s t-tests with multiple testing correction (Bonferroni), p-values in figure, all values < 0.05 shown. Black horizontal lines indicate median (**C-E**) or mean (**F**). Plots include all cells in the final integrated dataset, without additional filtering. (**G,H**) Per-cell mtDNA coverage of the WT (**G**) and mutant (**H**) allele (no depth cut-off applied). (**I**) Group size required to confidently identify mean heteroplasmy shifts at power >0.8 and p < 0.05. A mean group size of 179 would suffice to identify mean heteroplasmy shifts of 3.5-4%, assuming all cells shifted in the same direction. (**J**) Observed heteroplasmy distributions for *Tfam*, *Opa1* and *Polg* KO compared to simulated distributions generated by binomial downsampling of control cells to matched read depths (n = 5,000). Boxes 25^th^-75^th^ percentiles, mid-line median, whiskers extend to most extreme datapoints within 1.5 x IQR from hinges, all datapoints plotted. (**K**) Relationship between mtDNA depth and heteroplasmy variance. Single cells aggregated into read-depth bins of equal sample size to calculate local variance. Gray points without mtDNA depth reduction (all gRNAs excluding *Tfam*, *Polg*, and *Opa1*); coloured points represent *Tfam*, *Polg*, and *Opa1* perturbation groups. The gray dashed line represents the 95^th^ percentile fit from quantile regression on control cells (95% of control cells have technical variance at or below this line at a given depth). The gray ribbon represents bootstrapped 95% confidence intervals for the expected variance range modelled on the non-depleted population.

We next assessed how mtDNA CN and heteroplasmy level were affected by the genetic perturbation in each cell. Relative mtDNA CN was measured as the average single-cell mtDNA coverage from ATAC-seq reads^2,46^. Per-cell heteroplasmy level was quantified as the proportion of all reads matching the m.5024C>T mutant mtDNA molecule. The precision of per-cell heteroplasmy measurements was enhanced by combining reads across seven heteroplasmic single-nucleotide variants (SNVs) (Extended Data Fig. 4e–g), previously shown to be in *cis* or *trans* with the pathogenic m.5024C>T allele^47^, which we confirmed by calculating per-cell correlation of SNV heteroplasmy levels (Extended Data Fig. 4h,i) and long-read sequencing (Extended Data Fig. 4j,k). Per-cell heteroplasmy calls before and after mtDNA enrichment were highly correlated for all alleles (Extended Data Fig. 4f). When we visualized mtDNA coverage (Fig. 2c) and heteroplasmy levels (Fig. 2d) on a clustered UMAP plot (Fig. 2e), we noted that one cluster had markedly lower per-cell mtDNA content than the rest. This cluster had a distinct gene expression and accessibility profile compared to the other clusters and was enriched for gRNAs targeting *Tfam*, *Opa1* and *Polg*, with all other gRNA assignments underrepresented (Fig. 2f–i and Extended Data Fig. 5a,b). As expected^29,30,34^, measuring mtDNA CN in each perturbation group confirmed that KD of *Tfam*, *Opa1* and *Polg* caused a reduction in mean per-cell mtDNA read depth, with no concurrent change in nuclear ATAC-seq fragment counts (Fig. 2j and Extended Data Fig. 5c,d). This was accompanied by a decrease in mtDNA transcripts (Extended Data Fig. 5e). Each of the three gRNAs targeting these genes caused comparable mtDNA CN reduction, apart from one of the *Polg* gRNAs (Polg-6) that only slightly decreased *Polg* expression levels (Extended Data Fig. 4d; fold change = 0.97, *P* = 0.86) and, thus, had no impact on mtDNA CN (Extended Data Fig. 5d). We, therefore, removed this gRNA group from downstream analyses. None of the perturbations affected mean single-cell heteroplasmy levels, (Fig. 2k and Extended Data Fig. 5f–h), possibly reflecting limited power to detect changes of <~5% (Extended Data Fig. 5i).

Although there was no difference in mean heteroplasmy, we observed greater heteroplasmy variance in *Tfam*-KD cells compared to the NT gRNA group (*P* = 0.046, adjusted *P* = 0.87; Fig. 2l). Extending this analysis to also include cells with lower read depths (<20 across all heteroplasmic SNVs), resulted in a significantly higher heteroplasmy variance in *Tfam*-KD (adjusted *P* = 3.31 × 10^−22^), *Opa1*-KD (adjusted *P* = 6.76 × 10^−3^) and *Polg*-KD (adjusted *P* = 1.78 × 10^−8^) groups than in the NT group (Fig. 2m). To determine whether this increased variance was driven by stochastic sampling during mtDNA CN depletion, we modeled the expected variance using binomial sampling of control cells at the depths observed in each perturbation group, collectively spanning a wide range of mtDNA CN. Observed variances for each perturbation group were consistent with their corresponding expected variances, including for those exhibiting significant mtDNA CN reduction (adjusted *P* = 1 for *Tfam* and *Polg* KD, adjusted *P* = 0.308 for *Opa1* KD; Fig. 2n and Extended Data Fig. 5j). We also modeled the relationship between read depth and heteroplasmy variance on the basis of the population of cells from groups without mtDNA depth reduction (that is, all cells excluding *Tfam*, *Polg* and *Opa1* KD) (Extended Data Fig. 5k). Comparing this to the heteroplasmy variance in *Tfam*, *Polg* or *Opa1* KD showed similar depth-dependent trajectories in the nondepleted and the mtDNA-depleted populations (Extended Data Fig. 5k). Together, these results indicate that increased heteroplasmy variance may be explained by a perturbation-mediated genetic bottleneck through a reduction in mtDNA CN and read depth. Thus, in addition to technically validating MitoPerturb-Seq, our data indicate that mtDNA CN can be manipulated to modulate the range of heteroplasmy levels within single cells, potentially influencing the number of cells above or below the threshold required to cause an OXPHOS biochemical defect.

### mtDNA depletion affects nuclear gene expression

**Fig. 3:**
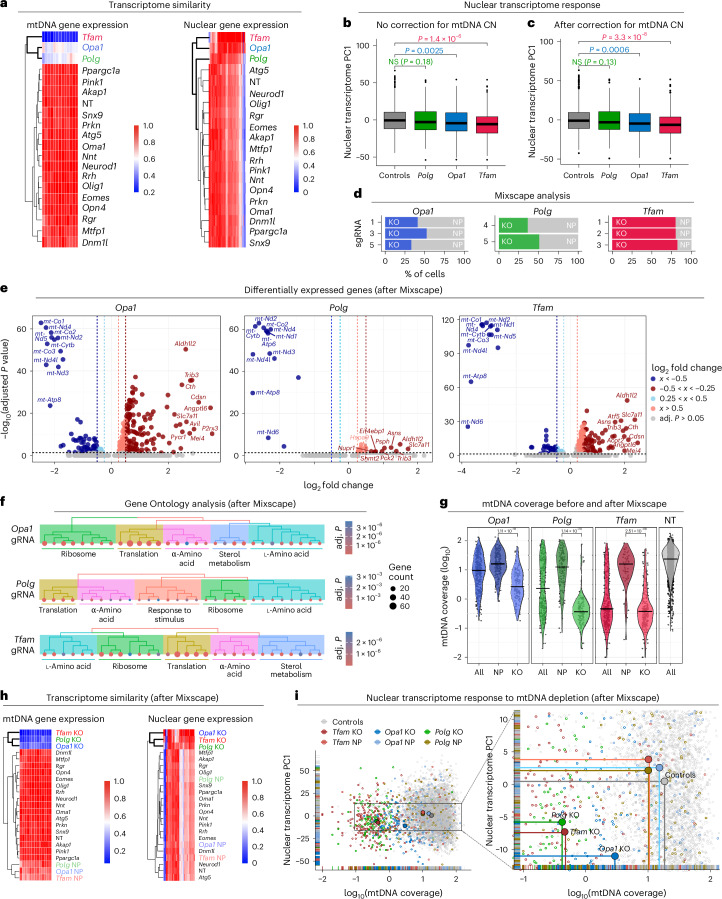
mtDNA depletion affects nuclear gene expression. **a**, Average gene expression of mtDNA (left) and nuclear (right) genes. Genes with zero expression were removed, filtered for adjusted *P* < 0.05 and scaled to the gene with highest expression. **b**,**c**, Distribution of PC1 of nuclear transcriptome across cells in each indicated perturbation group before (**b**) and after (**c**) correction for per-cell mtDNA coverage. A two-sided Wilcoxon signed-rank test with Benjamini–Hochberg correction for multiple comparisons was used to test for statistical differences. NS, not significant. Cell numbers per perturbation group are provided in Supplementary Table 3. Boxes encompass the 25th–75th percentiles, the center line indicates median and whiskers extend to the most extreme data points within 1.5× the interquartile range from the hinges; data points beyond this range are plotted as individual outliers. **d**, Percentage of cells assigned one of the three gRNAs targeting *Tfam* (red), *Opa1* (blue) and *Polg* (green) designated as KO following Mixscape analysis. The remaining cells were assigned as NP (gray). **e**, DEGs (adjusted *P* < 0.05) with a positive or negative log_2_ fold change of >0.25 (light colors) and >0.5 (dark colors) in *Opa1*-KO, *Polg*-KO and *Tfam*-KO cells compared to controls. The top ten upregulated and downregulated genes for each comparison are labeled. A two-sided Wilcoxon rank-sum test with Bonferroni correction for multiple comparisons was used to test for statistical differences. The full list of DEGs is provided in Supplementary Table 5. **f**, Significantly enriched GO terms, grouped by biological process, identified in the *Opa1*-KO, *Polg*-KO and *Tfam*-KO groups. A one-sided Fisher’s exact test with Benjamini–Hochberg correction for multiple comparisons was used to test for statistical differences. The full GO term analysis is provided in Supplementary Table 6. **g**, Distribution of mtDNA coverage (log_10_ transformed) in control cells and the *Opa1*, *Polg* and *Tfam* perturbed cells before and after assignment to KO and NP groups by Mixscape analysis. KO versus NP comparisons were conducted using pairwise two-sided Welch’s *t*-tests with multiple testing correction (Bonferroni). **h**, Average gene expression of mtDNA (left) and nuclear (right) genes, following Mixscape classification. As in **a**, genes were filtered for adjusted *P* < 0.05 and normalized to the most highly expressed gene. **i**, Strength of nuclear PC1 in all cells plotted against mtDNA coverage (log_10_ transformed), with *Opa1*, *Polg* and *Tfam* perturbation groups highlighted, separated by KO and NP cells after Mixscape.

**Extended Data Fig. 6:**
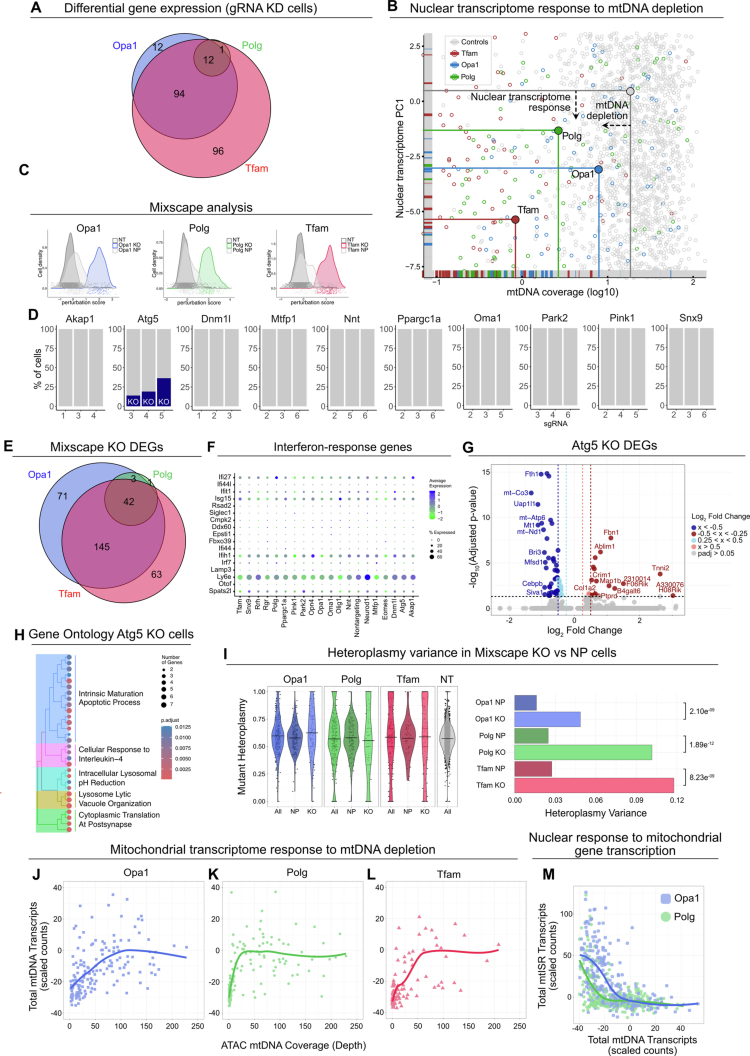
Differential gene expression upon gRNA perturbation. (**A**) Overlap of DEGs identified in *Tfam*, *Opa*1 and *Polg* perturbation groups. (**B**) Strength of principal component (PC1) of nuclear transcriptome in all cells plotted against mtDNA coverage (log10 transformed), with *Polg*, *Tfam* and *Opa1* perturbation groups and Controls highlighted, before Mixscape. (**C,D**) Perturbation scores of cells in the *Tfam*, *Opa1, Polg* gRNA groups (**C**) and proportion of cells assigned one of the three gRNAs targeting the indicated gene (**D**) following Mixscape. Remaining cells (grey) assigned as non-perturbed (NP). (**E**) Overlap of DEGs identified in the *Tfam*, *Opa1, Polg* perturbation groups after Mixscape. (**F**) Average expression of interferon-response genes in cells across the various perturbation groups, indicating overall low expression of these genes, and no activation. (**G**) Significantly differentially expressed genes (adjusted p-value < 0.05) with positive/negative log_2_ fold change of >0.25 (light colours) and >0.5 (dark colours) in *Atg5* KO cells compared to controls. Top 10 up- and down-regulated genes labelled. Two-sided Wilcoxon rank-sum test with Bonferroni correction for multiple comparisons. Full list of DEGs in Supplementary Table 5. (**H**) Significantly enriched Gene Ontology (GO) terms, grouped by biological process, identified in the *Atg5* KO group following Mixscape. One-sided Fisher’s exact test with Benjamini-Hochberg correction for multiple comparisons. Full details of identified GO terms are in Supplementary Table 6. (**I**) Distribution of mtDNA heteroplasmy (without mtDNA depth cut-off applied) in control cells and in *Opa1*, *Polg* and *Tfam* perturbation groups before and after assignment to KO and NP groups by Mixscape. The corresponding bar plot shows heteroplasmy variance in KO vs NP cells for each perturbation group. KO vs NP comparisons are Brown-Forsythe tests (one-way ANOVA) with multiple testing correction (Holm). (**J-L**) Per-cell mtDNA transcript levels against mtDNA coverage for *Opa1* (**J**), *Polg* (**K**) and *Tfam* (**L**) perturbation groups. (**M**) mtDNA transcript levels against combined transcript levels of genes involved in the mitochondrial integrated stress response in *Opa1* and *Polg* perturbation groups, indicating increased activation of the stress response in *Opa1* KO cells compared to *Polg* KO cells at similar mtDNA transcript levels.

A key feature of MitoPerturb-Seq is the ability to study transcriptional responses to mtDNA depletion at the single-cell level in an isogenic nuclear background. A priori, the mechanisms linking *Polg*, *Tfam* and *Opa1* KD with mtDNA depletion are likely to differ. *Polg* encodes the DNA polymerase responsible for mtDNA replication^29^, *Tfam* encodes a protein essential for mtDNA transcription and compaction^48^ and *Opa1* encodes a GTPase thought to have an indirect effect on mtDNA maintenance through regulation of mitochondrial membrane dynamics^34^. We, therefore, asked whether these pleiotropic effects had an impact on the downstream transcriptional response to mtDNA CN depletion. We identified 203 *Tfam*, 118 *Opa1* and 13 *Polg* differentially expressed genes (DEGs; log_2_ fold change > 0.25, adjusted *P* < 0.05), of which 107/215 (50%) were shared between at least two of the three groups and 12/215 (5.5%, all mtDNA-encoded) were shared among all three groups (Extended Data Fig. 6a and Supplementary Table 4). Interestingly, while mtDNA CN depletion (Fig. 2j and Extended Data Fig. 6b) and the reduction in mtDNA gene expression (Fig. 3a) caused by *Tfam* KD were more severe than upon *Opa1* or *Polg* KD, the nuclear gene expression profile of *Opa1*-KD cells was more similar to *Tfam*-KD than to *Polg*-KD cells (Fig. 3a,b and Extended Data Fig. 6b). The differences in nuclear transcriptomic response (nuclear first principal component (PC1)) were more prominent when we controlled for absolute mtDNA CN at the single-cell level (Fig. 3c), indicating that, at least for *Opa1*-KD cells, mtDNA depletion itself was only partially responsible for the nuclear transcriptomic response.

To focus our analysis on the most severely perturbed cells, we used Mixscape^49^ to define the difference between severely perturbed (knockout, KO) and less perturbed or nonperturbed (NP) cells within each gRNA group in an unbiased way (Fig. 3d and Extended Data Fig. 6c,d). Mixscape retained more *Tfam*-KO cells (219 out of 270, 81%) than *Opa1*-KO (124 out of 308, 40%) or *Polg*-KO (118 out of 286, 41%) cells, allowing the identification of 261 DEGs in *Opa1*-KO, 250 DEGs in *Tfam*-KO and 46 DEGs in *Polg*-KO cells (Fig. 3e and Supplementary Table 5). In total, 42 out of 325 (13%) DEGs were shared among all three KO groups and 190 out of 325 (58%) DEGs were shared between at least two of the three KO groups (Extended Data Fig. 6e). Gene Ontology (GO) analysis of DEGs showed a similar response in all three groups, with an upregulation of mitochondrial integrated stress response (mtISR) pathways, including genes involved in cytoplasmic protein translation, ribosome biogenesis and amino acid metabolism (Fig. 3f and Supplementary Table 6). Sterol metabolism genes were selectively depleted only in *Tfam*-KO and *Opa1*-KO but not *Polg*-KO cells (Fig. 3f). Enrichment of genes involved in cholesterol homeostasis has been reported in *Tfam*-KO mouse alveolar macrophages^50^, further confirming the physiological relevance of our MEF-based screening approach. However, interferon response genes, previously found to be activated upon *Polg* or *Tfam* deficiency^51–53^ or in heteroplasmic mice^54^ were not differentially expressed (Extended Data Fig. 6f). In the remaining perturbation groups, Mixscape analysis only detected KO cells in the *Atg5* gRNA group, indicating that perturbation of most target genes could not elicit a severe nuclear transcriptomic response in our conditions (Extended Data Fig. 6d). *Atg5* KO mainly affected pathways related to lysosome activity and cytoplasmic protein translation and stability (Extended Data Fig. 6g,h). All DEGs and pathways are provided in Supplementary Tables 5 and 6.

In the *Opa1*, *Polg* and *Tfam* gRNA groups, the KO cells retained by Mixscape had significantly stronger mtDNA depletion than NP cells, where mtDNA levels were similar to controls (Fig. 3g). There were no significant differences in mean heteroplasmy between KO and NP or control groups but we did observe significantly higher heteroplasmy variance in all three KO groups compared to NP cells (Extended Data Fig. 6i). mtDNA depletion was less marked in the *Opa1*-KO group (Fig. 3g), rendering the strongly perturbed *Polg*-KO cells more similar to *Tfam*-KO than to *Opa1*-KO cells (Fig. 3h,i). This is in keeping with the nuclear response to *Polg* or *Tfam* perturbation being primarily caused by mtDNA depletion, while other factors likely contribute to the response in *Opa1*-KD/KO cells. Focusing our analysis on mtDNA-encoded gene expression (mtRNA), *Opa1* KD caused a more severe decrease in mtRNA for the same level of mtDNA depletion than *Polg* KD (Extended Data Fig. 6j–m), supporting a role for *Opa1* in regulation of mtRNA transcription or stability, independent of its effect on mtDNA content, as previously suggested^55^.

**Extended Data Fig. 7:**
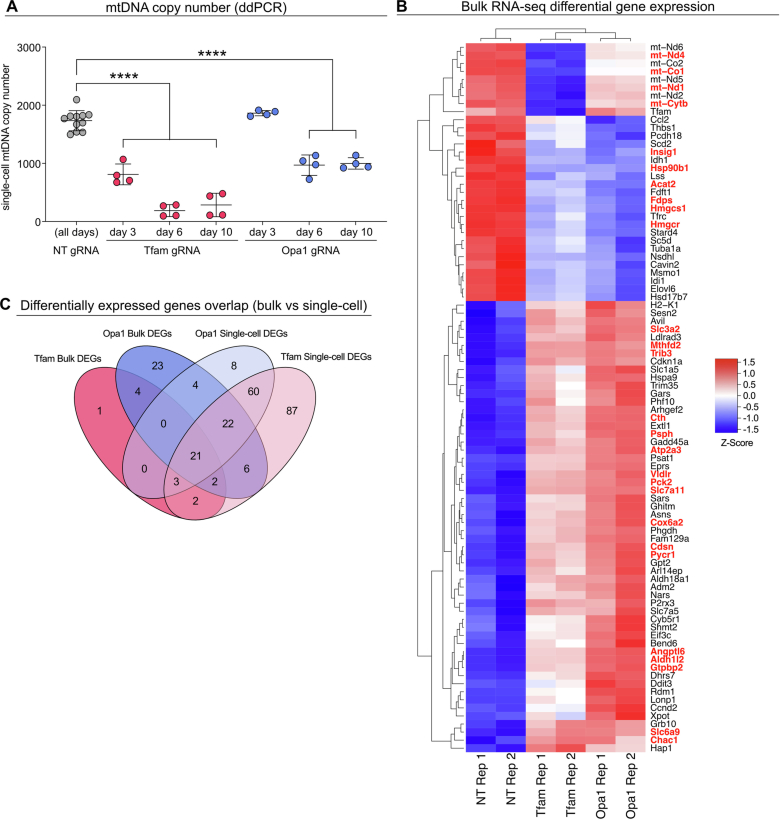
Bulk RNA-seq upon targeted perturbation of Tfam and Opa1. (**A**) Timecourse of mtDNA CN in m.5024 C > T MEFs (Clone 8 in Extended Data Fig. 1C, at 45% mean heteroplasmy) at 3 days, 6 days and 10 days following transduction with non-targeting (NT, grey), *Tfam* (red) or *Opa1* (blue) gRNAs. Each point represents a 20-cell bulk ddPCR measurement normalized to cell number (n = 11 measurements for NT gRNA and 4 measurements for all other groups). Data presented as mean values ± SD. One-way ANOVA with Tukey’s post-hoc test, pairwise comparisons to NT control, **** p < 0.0001. (**B**) Relative expression of DEGs in bulk RNA-seq data from *Tfam*- or *Opa1*-gRNA cells compared to non-targeting controls. All genes shown were significantly differentially expressed (FDR < 0.05 and log_2_-fold-change >0.25) in at least one gRNA condition; genes highlighted in red were differentially expressed in both *Tfam*- and *Opa1*-gRNA cells. (**C**) Overlap of DEGs identified in bulk RNA-seq and MitoPerturb-Seq *Tfam* and *Opa1* KD cells. Source data

We independently validated these findings by performing bulk RNA-seq analysis and droplet digital PCR (ddPCR)-based mtDNA CN measurements after single-gRNA transduction using a second m.5024C>T clone (clone 8, 45% heteroplasmy; Extended Data Fig. 1c). *Tfam* KD resulted in a rapid and severe mtDNA CN reduction 3 days after transduction (Extended Data Fig. 7a), implicating active mtDNA degradation, which is in keeping with TFAM having a crucial role in nucleoid compaction and protecting mtDNA from lysosomal or mitochondrial nuclease-mediated degradation^56–58^. Nevertheless, the transcriptomic response to *Opa1* KD 6 days after gRNA transduction was more severe than to *Tfam* KD (82 and 33 DEGs, respectively, at false discovery rate (FDR) < 0.05; overlap of 27 genes) (Extended Data Fig. 7b,c). Together, these data indicate that, although *Opa1*, *Polg* and *Tfam* perturbation all activate a similar set of mtISR genes, retrograde mitochondrial–nuclear signaling may occur through different perturbation-specific pathways, with cells responding to *Opa1* KD independent of mtDNA depletion, in line with OPA1’s upstream role in mitochondrial homeostasis and membrane dynamics^59^. The level of mtDNA depletion (more severe in *Tfam* KO than *Opa1* KO) and strength of the nuclear transcriptional response (stronger in *Opa1* KO than *Tfam* KO) was consistent between both MEF clones, each carrying different heteroplasmy levels, suggesting that, at least in this context, the cellular response to these perturbations was independent of the heteroplasmy level and occurred well below the biochemical threshold of the m.5024C>T mutation^25^. Critically, simultaneously probing these mechanisms in nuclear isogenic cells, grown together and exposed to the same environment, allowed us to avoid additional and potentially unknown confounders impacting cellular stress responses.

### ATF4 only partially contributes to the response to mtDNA depletion

**Fig. 4:**
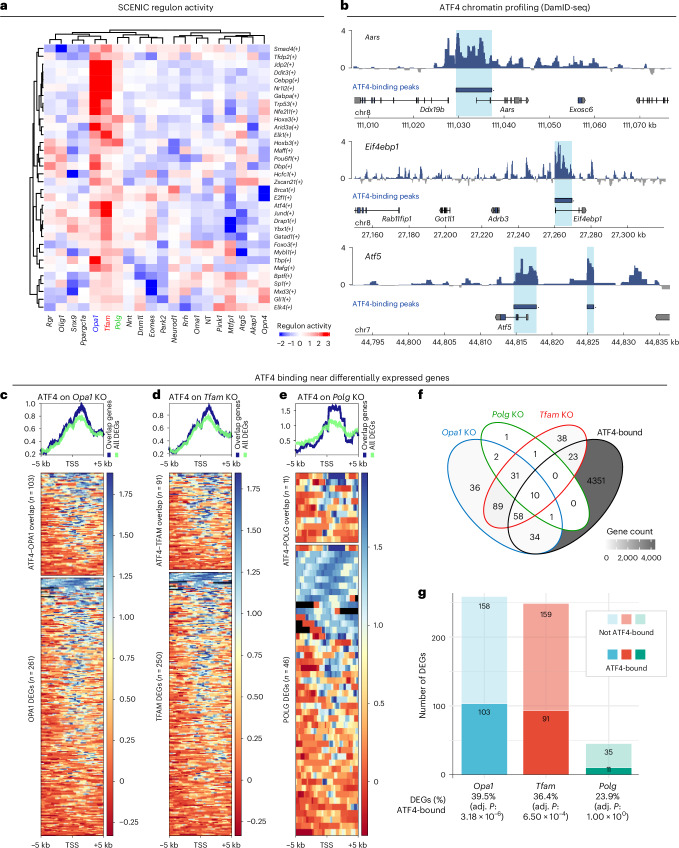
ATF4 only partially contributes to the response to mtDNA depletion. **a**, Regulon activity (AUC > 0) for three selected perturbation groups (*Opa1*, *Polg* and *Tfam*) following SCENIC analysis. **b**, ATF4 DamID-seq profiles in heteroplasmic MEFs across three ATF4 target genes (*Aars*, *Eif4ebp1* and *Atf5*). ATF4-binding peaks are shaded light blue. **c**–**e**, Metagene plots (top) and heat maps (bottom) showing ATF4 DamID-seq signal across TSSs of all genes differentially expressed in *Opa1* (**c**), *Tfam* (**d**) or *Polg* (**e**) perturbation groups (bottom, green), or only those DEGs associated (top, blue) with nearby ATF4-binding sites. **f**, Overlap between ATF4 DamID-seq target genes and DEGs in each of the indicated perturbation groups. **g**, Number of DEGs (total bar height) and the number and percentage bound by ATF4 DamID-seq (dark shading). A one-sided Fisher’s Exact test with Bonferroni correction was used to test significance differences across all unique genes detected in gRNA-containing cells.

**Extended Data Fig. 8:**
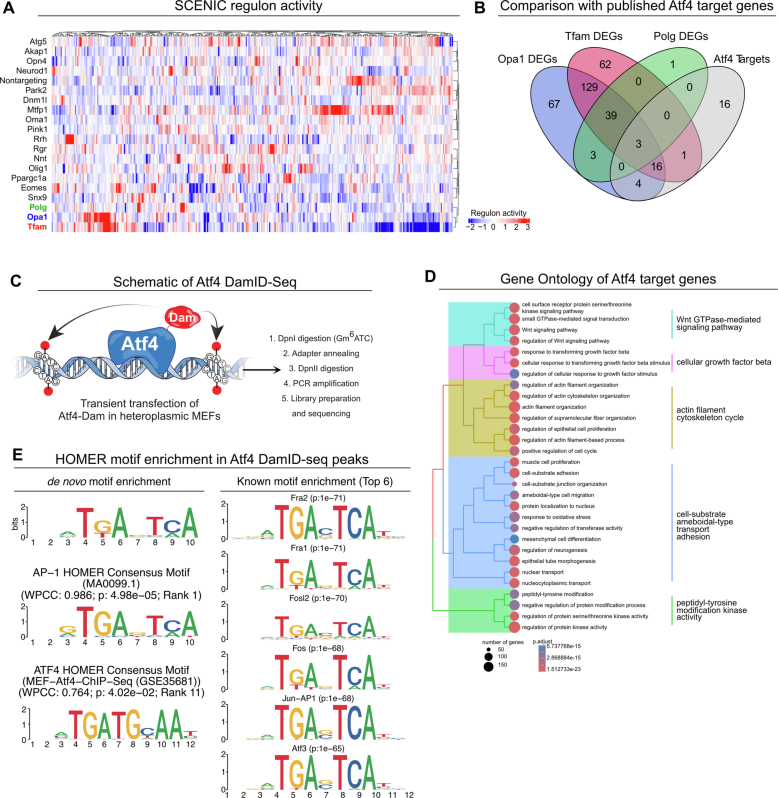
ATF4 DamID-seq in heteroplasmic MEFs. (**A**) Regulon activity following SCENIC analysis. Average AUC scores were calculated for each gRNA group; all regulons identified are shown and also listed in Supplementary Table 7. (**B**) Intersection of *Tfam*, *Opa1* and *Polg* KO MitoPerturb-Seq DEGs (post-Mixscape analysis) with a published list of high-confidence ATF4 target genes. (**C**) Schematic of ATF4-DamID-seq in heteroplasmic MEFs. Adapted, with permission, from ref. ^87^., under a Creative Commons Attribution 4.0 International License. (**D**) Significantly enriched Gene Ontology (GO) terms, grouped by biological process, identified in the ATF4 target genes. One-sided Fisher’s exact test with Benjamini-Hochberg correction for multiple comparisons. (**E**) Motif enrichment analysis of ATF4 DamID-seq peaks. *De-novo* motif discovery (left) identified a top-ranked motif matching the AP-1 consensus (Fos::Jun; Rank 1) as well as the canonical ATF4 motif in the HOMER database, representative of ATF4-CEBPG heterodimer binding (Rank 11). Known motif enrichment (right) shows high prevalence of AP-1 family members. P-values denote significance of enrichment; WPCC, Weighted Pearson correlation coefficient.

To define the factors driving the transcriptional response to mtDNA depletion in *Tfam*-KD, *Opa1*-KD and *Polg*-KD cells, we performed single-cell regulatory network inference and clustering (SCENIC) analysis^60^. SCENIC allows inference of gene regulatory networks (regulons) that show differential activity in each of our perturbation groups (Extended Data Fig. 8a and Supplementary Table 7). We identified 34 regulons that were differentially active in all three KD groups (Fig. 4a), including regulons driven by the transcription factors ATF4, DDIT3, CEBPG or JUND, known to be involved in the cellular response to stress in cell culture^61^, and in *Tfam*-KO or *Opa1*-KO mice^62,63^. Comparing our dataset to a recent systematic review of putative ATF4 target genes^61^, 24 out of 40 (60%) high-confidence ATF4-responsive protein-coding genes were also differentially expressed in at least one of our *Tfam*-KO, *Opa1*-KO and *Polg*-KO groups (Extended Data Fig. 8b). To independently validate these findings, we used DamID-seq^64,65^ for genome-wide profiling of ATF4 chromatin-binding sites in our heteroplasmic MEFs (Fig. 4b and Extended Data Fig. 8c). This identified 5,789 ATF4-binding peaks near 4,477 genes (Supplementary Tables 8 and 9 and Extended Data Fig. 8d). The top de novo motif identified by motif enrichment analysis had a high similarity to the canonical AP1 consensus motif (FOS::JUN, JASPAR MA0099.1; weighted Pearson’s correlation coefficient = 0.986, *P* = 4.98 × 10^−5^). This was confirmed by an analysis of known motifs, where the most highly enriched motifs were AP1 family members that bind the same 5′-TGA[GC]TCA-3′ consensus, suggesting ATF, JUN and FOS co-occupancy of these ATF4 DamID-seq binding sites^66^. The canonical ATF4 (bZIP) motif in the HOMER database, 5′-MTGATGCAAT-3′, characteristic of ATF4–CEBPG heterodimer binding^67^, was also highly enriched (rank 11, *P* = 1 × 10^−17^) (Extended Data Fig. 8e), validating DamID-seq peaks as genuine ATF4-binding sites. Of the 325 DEGs in *Tfam*-KO, *Polg*-KO and *Opa1*-KO cells, 126 (38.8%) were near ATF4-binding sites in heteroplasmic MEFs, with binding mostly near transcription start sites (TSSs) (Fig. 4c–e). However, at our current thresholds (FDR < 0.05; log_2_-transformed fold change > 0.25), the majority of DEGs upon *Opa1* KO (158 out of 261, 60.5%), *Tfam* KO (159 out of 250, 63.6%) or *Polg* KO (35 out of 46, 76.1%) were not ATF4 bound (irreproducible discovery rate (IDR) < 0.01; Fig. 4f,g), indicating an important but not exclusive role for ATF4 in responding to mtDNA-related stress. Critically, a range of other non-ATF4 transcription factor regulons were found upon SCENIC analysis (Fig. 4a). These are provided for further exploration in Supplementary Table 7 and are likely to coregulate the response to perturbation of these mtDNA maintenance genes or to mtDNA depletion in heteroplasmic cells.

### mtDNA depletion delays cell-cycle progression across all stages

**Fig. 5:**
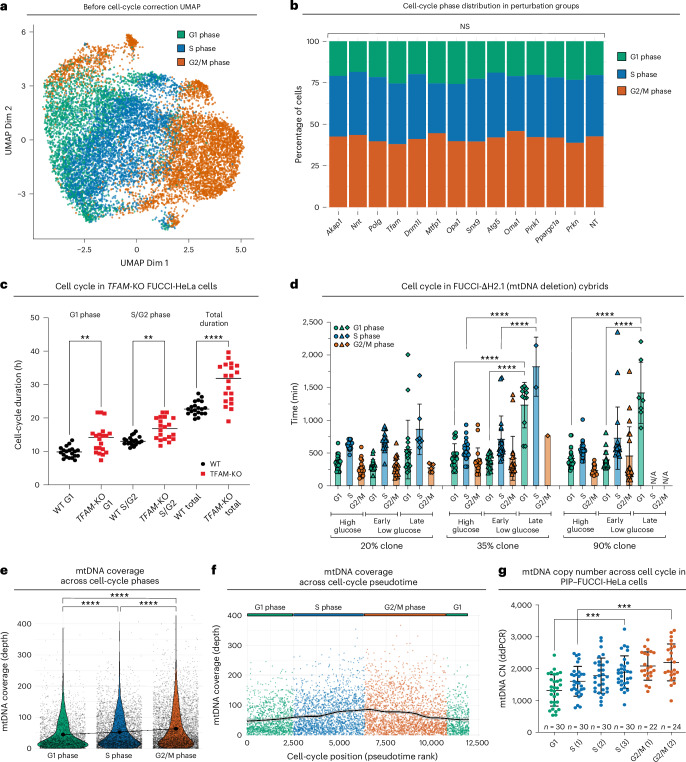
mtDNA depletion delays cell-cycle progression and relaxed replication. **a**, UMAP clustering based on RNA-seq before regression of cell-cycle heterogeneity. Cells are colored by cell-cycle phase. **b**, Proportion of cells in each perturbation group assigned to each cell-cycle stage. Two-sided chi-squared tests with multiple testing correction (Benjamini–Hochberg) were used to test for significant differences. **c**, Cell-cycle phase duration times in FUCCI-expressing WT and *TFAM*-KO HeLa cells. Phase duration was calculated across one full cycle (G1 and S/G2) for 20 cells per condition. Comparisons were conducted using two-tailed unpaired *t*-tests. **P < 0.01 and ****P < 0.0001. Data are presented as the mean ± s.d. **d**, Cell-cycle phase duration in ΔH2.1 mtDNA deletion cybrid clones stably expressing PIP–FUCCI, cultured in high-glucose or low-glucose medium and imaged every 20 min over a period of 72 h. Phase duration was calculated across one full cycle (G1, S and G2/M) for 20 cells (early, first 24 h; late, after 24 h). Data are presented as the mean ± s.d. One-way ANOVAs were performed per cell-cycle phase for each clone with Tukey’s post hoc test. ****P < 0.0001. All significant pairwise comparisons are shown. N/A, no observed cells progressed to this phase during the experiment. **e**, mtDNA coverage across Seurat cell-cycle phases. A one-way ANOVA with Tukey’s post hoc test was used to test for significant differences. ****P < 0.0001. **f**, mtDNA coverage across cell-cycle pseudotime with cells ranked on the basis of cell cycle pseudotime (tricycle *π*) score. The fit line is a smoothed locally estimated scatterplot smoothing regression (span = 0.2). Shaded regions around the fit line indicate the 95% CI. **g**, mtDNA CN in WT PIP–FUCCI-HeLa cells flow-sorted by cell-cycle phase (sorting strategy in Extended Data Fig. 9e). Cell numbers per group are indicated on the graph. Data are presented as the mean ± s.d. A one-way ANOVA with Tukey’s post hoc test was used to test for significant differences. ***P < 0.001. Source data

**Extended Data Fig. 9:**
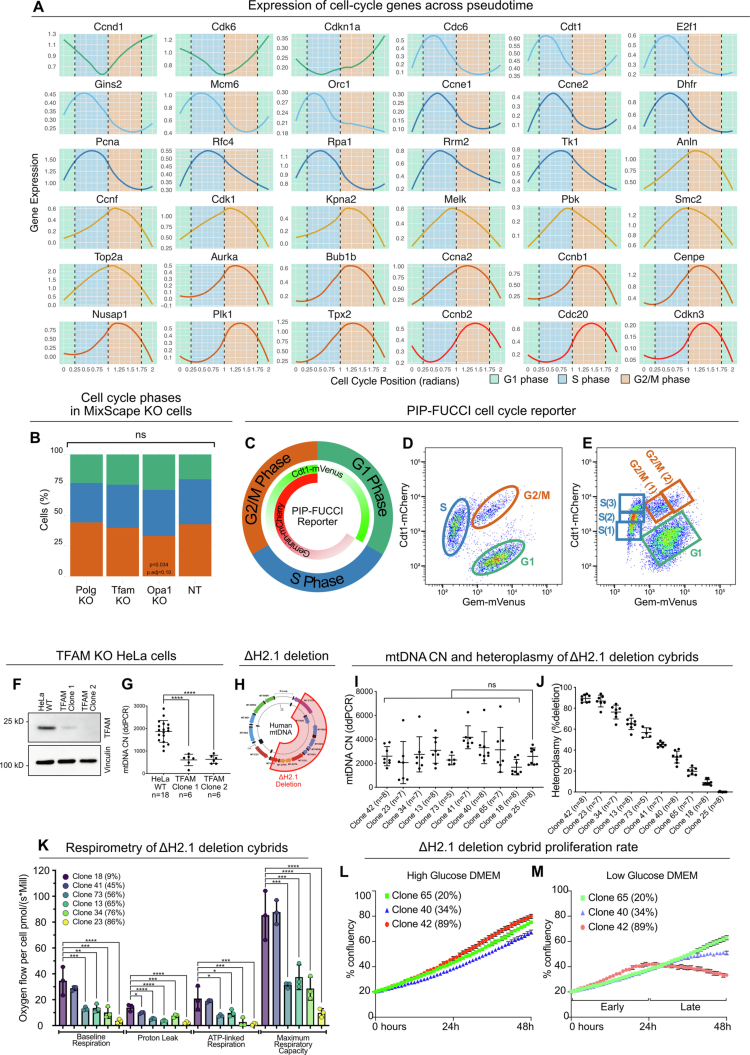
Cell cycle slowing in response to mtDNA depletion. (**A**) Expression profiles of indicated cell-cycle genes across cell-cycle pseudotime determined by tricycle. (**B**) Percentage of cells in each cell-cycle phase in *Tfam*, *Opa1*, *Polg* KO cells, post-Mixscape, compared to non-targeting gRNA cells. Chi-squared tests with multiple testing correction (Bonferroni); ns, not significant. (**C**) Schematic showing the expression patterns of the PIP-FUCCI fluorescent reporters Cdt1-mVenus and Geminin-mCherry across the cell-cycle. (**D,E**) Gating strategies used to isolate cells for mtDNA CN measurements based on cell-cycle phase for both inter-phase (**D**) and intra-phase (**E**) comparisons. (**F,G**) Western blot (**F**) and ddPCR measurements (**G**) showing KD of TFAM protein (**F**) and reduced mtDNA CN (**G**) in HeLa cells transduced with two independent *TFAM* gRNAs. Vinculin is shown as a loading control (**F**). Number of individual cells per group is indicated in (**G**), data presented as mean values ± SD. One-way ANOVA with Tukey’s post-hoc test, **** p < 0.0001. (**H**) Map of the human mtDNA, highlighting the 7.5 Kb deletion present in the DeltaH2.1 cybrid cell line. (**I,J**) mtDNA CN (**I**) and heteroplasmy (**J**) of eight clonal populations isolated from the DeltaH2.1 cybrid cell line. Cell numbers per group indicated on graphs. Data presented as mean values +/- SD. One-way ANOVA of the mtDNA CN distributions was significant (p = 0.015); pairwise comparisons of all heteroplasmic clones to Clone 25 (0% heteroplasmy) using Tukey’s post-hoc test; ns, not significant. (**K**) Respirometry of DeltaH2.1 cybrid cells with increasing heteroplasmy levels, using Oroboros, showing baseline respiration (BR), proton-leak (PL), ATP-linked respiration (AR) and maximal respiratory capacity (MC). Three separate assays per clone. Data presented as mean values ± SD. One-way ANOVA with Tukey’s post-hoc test performed for each tested parameter, all significant pairwise comparisons are shown. * p < 0.05, ** p < 0.01, *** p < 0.001, **** p < 0.0001. (**L,M**) Growth curves of DeltaH2.1 cybrid clones carrying different levels of heteroplasmy in high (**L**) and low (**M**) glucose culture medium. Confluency values averaged over 8 individual images per timepoint for each clone in each condition, data presented as mean values ± SEM. Figure created in BioRender. Van den Ameele, J. (2026) https://BioRender.com/j3ro7vq. Source data

Having identified the transcriptomic changes caused by mtDNA depletion, we next asked how this could impact cellular physiology and behavior. Cell proliferation is a sensitive indicator of mitochondrial activity, with OXPHOS dysfunction previously shown to cause cell-cycle slowing primarily at the G1/S transition^68–70^. We performed cell-cycle stage annotation on our MitoPerturb-Seq dataset using Seurat^71^ (Fig. 5a) and continuous cell-cycle pseudotime analysis^72^ (Extended Data Fig. 9a). UMAP analysis of heteroplasmic MEFs before cell-cycle correction showed cells mainly segregating on the basis of cell-cycle stage (Fig. 5a). To our surprise, we saw no significant differences in the proportion of cells in each cell-cycle phase between the perturbation groups (Fig. 5b). After Mixscape analysis, only *Opa1*-KO but not *Tfam*-KO or *Polg*-KO cells showed a slight (*P* = 0.034, adjusted *P* = 0.10) increase in the proportion of cells in G1 phase compared to control cells (Extended Data Fig. 9b). This indicates that, in contrast to previous reports describing cell-cycle slowing specifically at the G1/S transition upon OXPHOS dysfunction^68–70^, there was no selective delay in G1/S progression in *Tfam*-KO and *Polg*-KO cells, despite severe mtDNA depletion.

To validate these findings, we generated transgenic cell lines expressing a fluorescent ubiquitination-based cell-cycle indicator (FUCCI)^73^ (Extended Data Fig. 9c–e and Supplementary Video 1) and analyzed cell-cycle duration in WT and *TFAM*-KO FUCCI-HeLa cells (Extended Data Fig. 9f,g), and in FUCCI transgenic cybrid cells carrying a heteroplasmic large-scale mtDNA deletion encompassing the major arc^74^ (FUCCI-DeltaH2.1; Extended Data Fig. 9h). *TFAM*-KO FUCCI-HeLa cells had severe mtDNA depletion (Extended Data Fig. 9g) and displayed an increase in overall cell-cycle length compared to WT FUCCI-HeLa cells, caused by increased duration of both G1 and S/G2 phases (Fig. 5c). These findings in *TFAM*-KO FUCCI-HeLa cells are consistent with our MitoPerturb-Seq data from heteroplasmic *Tfam*-KO (and *Polg*-KO) MEFs, where a consistent delay across all stages of the cell cycle would not change the proportion of cells in each individual phase. Clones of FUCCI-DeltaH2.1 cells had no mtDNA depletion (Extended Data Fig. 9i) but carried a range of heteroplasmies (Extended Data Fig. 9j), with higher heteroplasmy severely impacting OXPHOS activity (baseline respiration, ATP-linked respiration and maximum respiratory capacity) (Extended Data Fig. 9k). In contrast to *TFAM*-KO cells, FUCCI-DeltaH2.1 clones with low (20%), mid (35%) and high (90%) heteroplasmy all proliferated normally (Extended Data Fig. 9l). However, the proliferation rates of cells with mid and high (but not low) heteroplasmy decreased when cultured in low-glucose medium (Extended Data Fig. 9m). This was mainly caused by an increase in G1 phase duration, with many cells with mid and high heteroplasmy not progressing from G1 to S phase when cultured in low-glucose medium for > 48 h (Fig. 5d). Together, these data indicate differential requirements and sensing of mtDNA abundance versus OXPHOS activity to sustain cell-cycle progression, with the G1/S transition being most sensitive to heteroplasmy and OXPHOS deficiency. However, when confronted with severe mtDNA depletion, as in *Tfam*-KO cells, cells slow cell-cycle progression across all phases.

### Relaxed replication of mtDNA across the cell cycle

**Extended Data Fig. 10:**
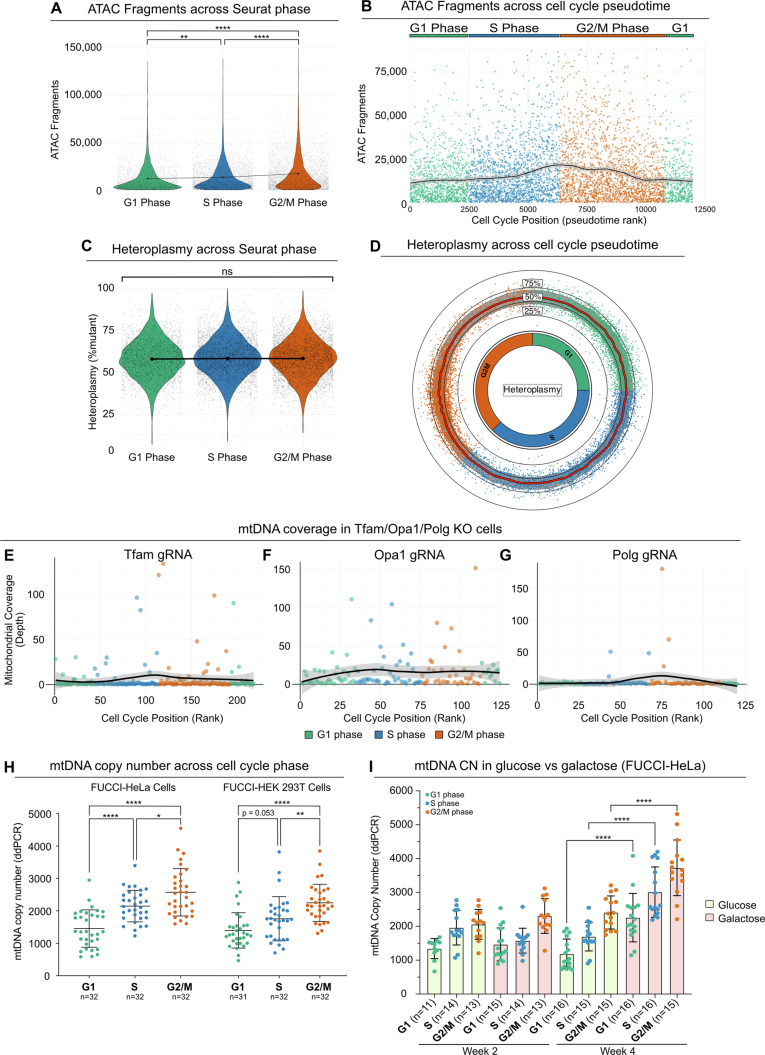
Relaxed replication of mtDNA. (**A,B**) Nuclear ATAC fragment counts across cell cycle stages using Seurat (**A**) or across cell cycle pseudotime based on tricycle pi score (**B**). Fit line for (**B**) is a smoothed LOESS regression (span = 0.2), shaded regions around the fit line indicate 95% CI. One-way ANOVA with Tukey’s post-hoc test, ** p < 0.01, **** p < 0.0001. (**C,D**) Heteroplasmy levels across cell cycle stages using Seurat (**C**) or across cell cycle pseudotime based on tricycle pi score (**D**). Red line = mean, grey band = standard deviation. One-way ANOVA with Tukey’s post-hoc test; ns, not significant. (**E-G**) mtDNA coverage across cell cycle pseudotime with cells ranked based on tricycle pi score for the *Tfam* (**E**), *Opa1* (**F**) and *Polg* (**G**) perturbation groups. Fit lines are smoothed LOESS regressions (span = 0.7), shaded regions around the fit lines indicate 95% CI. (**H**) Single-cell mtDNA CN measurements in PIP-FUCCI-expressing HeLa and HEK293T cells following flow sorting based on cell cycle phase. Cell numbers per group are indicated on graph. Data presented as mean values ± SD. One-way ANOVA with Tukey’s post-hoc test performed across cell-cycle phases for each cell type, * p < 0.05, ** p < 0.01, **** p < 0.0001. (**I**) Single-cell mtDNA CN measurements in PIP-FUCCI-expressing HeLa cells following flow sorting based on cell cycle phase. Cells cultured in DMEM supplemented with either high-glucose or galactose for four weeks. Cell numbers per group indicated on graph. Data presented as mean values ± SD, one-way ANOVA with Tukey’s post-hoc test per cell-cycle phase across time points and culture conditions, **** p < 0.0001. Source data

During each cell cycle, the nuclear DNA is replicated only once, in a strictly regulated process during the S phase. In contrast, the mtDNA is thought to undergo relaxed replication, independent of the cell cycle^75,76^. Previous studies found conflicting evidence of whether mtDNA replication rates differ across cell-cycle stages but these lacked temporal resolution, relied on indirect mtDNA CN measurements or were based on cell-cycle synchronization, which directly impacts mitochondrial and cellular metabolism^77–80^. We reasoned that combined RNA and mtDNA profiling from a homogeneous population of proliferating cells, as in our MitoPerturb-Seq dataset, could provide an alternative, unbiased approach to characterize cell-cycle-related mtDNA dynamics at the single-cell level. Analysis of mtDNA scATAC-seq read counts across the cell cycle in MEFs showed a progressive increase in mtDNA coverage across G1/S and S/G2/M stages (Fig. 5e), as well as across continuous cell-cycle pseudotime^72^ (Fig. 5f), in keeping with mtDNA replication being fully ‘relaxed’ (ref. ^75^). Crucially, this also further validates our approach to use ATAC-seq read coverage as a proxy for mtDNA CN, allowing detection of subtle changes in mtDNA content within single cells. Nuclear ATAC-seq read count also increased across cell-cycle phases but most strongly during the S/G2 transition (Extended Data Fig. 10a,b). Heteroplasmy levels remained constant between cell-cycle phases (Extended Data Fig. 10c) and over cell-cycle pseudotime (Extended Data Fig. 10d). The mtDNA depletion caused by *Tfam*, *Polg* or *Opa1* KO was also present in all cell-cycle stages and across cell-cycle pseudotime (Extended Data Fig. 10e–g). To validate these findings, we measured absolute mtDNA CN by single-cell ddPCR^81^ in WT FUCCI-HeLa and FUCCI-HEK293T cell lines (Extended Data Fig. 10h,i). This indeed revealed a linear increase in mtDNA CN, not only across cell-cycle phases (G1, S and G2/M) (Extended Data Fig. 10h) but also across the early, mid and late S phase and between the early and late G2 phase (Fig. 5g and Extended Data Fig. 9e). Interestingly, culturing WT FUCCI-HeLa cells in galactose medium to force respiration through OXPHOS induced an increase in mtDNA CN, whilst maintaining a linear increase across cell-cycle stages (Extended Data Fig. 10i), indicating that cells actively adjust mtDNA CN in response to higher dependence on mitochondrial ATP production. Our findings, thus, validate previous work at high temporal resolution, demonstrating fully relaxed replication of WT and mutant mtDNA molecules across the cell cycle but modulated in response to bioenergetic requirements.

## Discussion

Single-cell mtDNA analysis in healthy humans^17^ or in mice and persons with inherited or age-related mtDNA mutations^18,19^ has found an extraordinary degree of mosaicism, with virtually all cells across tissues and organisms containing different abundance and mixtures of WT and mutant mtDNA. Although the phenotypes caused by these mtDNA mutations are inherently mosaic at the cellular level, most studies to date have relied on bulk, tissue-wide analysis, averaging out the single-cell consequences of mtDNA-related heterogeneity and, thus, limiting the sensitivity to detect disease-relevant phenotypes and mechanisms. Technical challenges when perturbing and measuring mtDNA at the single-cell level mean that most studies to date have been correlative and little is known about which nuclear-encoded proteins respond to or regulate changes in mtDNA CN and mutation load in individual cells or cell types.

Building on single-cell CRISPR screening methods^22^ and simultaneous scRNA-seq and mtDNA-seq^24,82^, we developed MitoPerturb-Seq for high-throughput unbiased forward genetic screening of factors with a direct causal effect on mtDNA CN and heteroplasmy within individual cells. As proof of concept, we screened a panel of 60 gRNAs, targeting 13 nuclear-encoded genes known to be involved in mtDNA dynamics, in Cas9-transgenic MEFs from mice carrying a heteroplasmic mtDNA tRNA^Ala^ mutation corresponding to a human pathogenic variant^25,27^. Perturbation of three of these genes, *Tfam*, *Opa1* and *Polg*, caused strong single-cell mtDNA depletion, with large overlap in the single-cell transcriptomic response to each perturbation, driven by comparable transcription factor regulons. One of these transcription factors, ATF4, has been studied extensively as a central factor mediating the response to mitochondrial dysfunction and cellular stress^83–85^. However, in our data and conditions, only approximately one third of DEGs were directly bound by ATF4, which, together with our regulon analysis, indicaes that many other factors are involved. We provide a ranked resource of transcription factors involved in modulating mtDNA CN, including established mtISR genes, such as two small MAF transcription factors, MAFF and MAFG, known to be involved in tissue-specific responses to oxidative stress^86^. Cell-type-specific expression of stress-responsive transcription factors, together with differential chromatin accessibility^87^, is likely to underlie tissue-specific and context-dependent resilience and vulnerability to mitochondrial dysfunction^88^; hence, it will be important to extend our approach to other cell types.

We conducted our experiments in heteroplasmic MEFs and targeted some of the few nuclear-encoded genes that were previously found to regulate heteroplasmy levels in other contexts^10,12,13,28–30,32,39,41^. Nevertheless, we could not detect significant changes in heteroplasmy levels in any of the perturbation groups, with several groups also showing no significant transcriptional response to target gene KO. Although technical considerations, including those inherent to pooled single-cell CRISPR screens^89^ (low target gene expression in MEFs, cell-to-cell heteroplasmy variability and short duration of perturbation), may underlie this lack of effect to some extent, many of these genes will have context-dependent and cell-type-specific or organism-specific functions. Future experiments including more cells, different cell types, higher mtDNA read depth and longer timeframes will be required to confidently reveal or exclude genes and processes that modulate heteroplasmy levels, although our findings indicate that even severe gene KD will have very subtle effects at best.

How nuclear and mtDNA replication rates are coordinated during cell growth and proliferation remains poorly understood^76–78^. We provide further evidence for completely relaxed mtDNA replication at the single-cell level, independent of cell-cycle stages, in a range of WT, mtDNA-depleted, homoplasmic and heteroplasmic human and mouse cell lines. Interestingly, cell-cycle slowing in response to mtDNA depletion affects the entire cell cycle, equally delaying all phases. This differs from the G1/S-specific delay in response to high heteroplasmy levels or targeted OXPHOS dysfunction that we and others have observed^68–70^ and might indicate the presence of a specific, yet unknown nuclear sensing mechanism to coordinate cell-cycle progression across all cell-cycle stages with mtDNA abundance or replication.

Our targeted library provides proof of principle for completely unbiased discovery through genome-wide CRISPR screening. In addition, PCR-based and hybridization-capture-based targeted enrichment approaches allow selective sequencing of gRNAs and mtDNA, potentially reducing cost and increasing throughput >5-fold. Moreover, MitoPerturb-Seq is uniquely placed to conduct screening in disease-relevant postmitotic cell types, including in vivo^90^ or in organoids derived from human induced pluripotent stem cells. This is particularly relevant, as mtDNA mutations mostly accumulate in and affect postmitotic cells^4,88^. The recent advent of mtDNA-modifying technologies to engineer mtDNA point mutations^91,92^ and deletions^32,46^ in any cell or model system means that MitoPerturb-Seq can now be used to compare the response to perturbation in isogenic cells with a range of heteroplasmic and homoplasmic mtDNA defects. We envisage this to be combined with other functional measurements, such as flow-cytometry-based mitochondrial membrane potential measurement^93^, and with more affordable combinatorial indexing approaches, such as SHARE-seq^94^, for high-throughput simultaneous detection of gRNAs, mtDNA CN and heteroplasmy in millions of heterogeneous cells from mosaic cultures, tissues and organisms. Increased throughput will also allow combinatorial screening using dual-gRNA vectors to investigate genetic interactions by probing the effects of perturbing more than one gene simultaneously^95^. In conclusion, MitoPerturb-Seq provides a powerful forward genetic screening approach to discover the biological mechanisms driving age-dependent mtDNA CN reduction or clonal expansion of damaging mtDNA mutations, thereby uncovering novel druggable targets to treat both rare and common mtDNA-related and neurodegenerative disorders.

## Methods

### Animal models and husbandry

Animals were housed in a facility according to the UK Home Office guidelines upon approval by the University of Cambridge Animal Welfare and Ethical Review Body and the UK Home Office (project license P6C97520A). Mice were kept in individually ventilated cages at 20–24 °C, 45–65% humidity on a 12-h light–dark cycle. The mouse line used in this study was m.5024C>T (allele symbol: *mt-Ta*^m1Jbst^, MGI 5902095), bred on the C57BL/6 background under protocol 5 of project license P6C97520A (Breeding and Maintenance of Genetically Modified Animals).

### Cell culture and transgenic cell lines

Unless otherwise stated, all cells in this study were maintained in high-glucose DMEM with pyruvate (Gibco, 41966-029) supplemented with 10% FBS (Gibco, 16000-044) and 50 μg ml^−1^ uridine (Sigma, U3750) at 37 °C and 5% CO_2_ in a humidified incubator. WT HeLa cells were purchased from the European Collection of Authenticated Cell Cultures (93021013). HEK293T cells were purchased from Takara (Lenti-X 293T cells; 632180). None of the cell lines used in this study appear in version 13 of the ICLAC Register of Misidentified Cell Lines.

Primary m.5024C>T MEFs were described previously^11^, isolated from individual E13.5 embryos and immortalized by transfection with the SV40 large T antigen (pBSSVD2005, a gift from D. Ron; Addgene, plasmid 21826). Cas9-expressing m.5024C>T MEFs were generated by transducing with lentiCas9-Blast lentivirus (Addgene, 52962)^96^. Following transduction, cells were selected with 10 μg ml^−1^ blasticidin (Sigma, SBR00022). Clonal populations were isolated and heteroplasmy levels assessed by pyrosequencing. To test Cas9 efficiency, m.5024C>T Cas9-blast clones were first transduced with pLJM1-EGFP lentivirus (Addgene, 19319)^97^ to stably express eGFP and then transduced with CROPseq–RFP lentivirus expressing eGFP-targeting gRNAs^98^. Editing efficiency was assessed by flow cytometry, with mRFP expression (that is, integration and expression of the gRNA cassette) in absence of eGFP expression (that is, KO of the transgene) indicating successful editing.

*TFAM*-KO HeLa cell lines were generated using gRNAs and lentivirus described below. Following transduction, cells were selected for transgene expression with 1 μg ml^−1^ puromycin (Sigma, P4512). To isolate clonal *TFAM*-KO populations, puromycin-selected cells were subcloned, and *TFAM* KO was confirmed by western blot. PIP–FUCCI transgenic cells were generated using plasmids and lentivirus described below. Following transduction, cells were subcloned, selecting cells on the basis of the Cdt1–mVenus G1-phase reporter. Clones were checked after expansion by flow cytometry to identify clones showing expected mVenus and mCherry expression patterns for use in downstream experiments.

ΔH2.1 cybrid cells carrying a heteroplasmic mtDNA deletion were obtained from C. Moraes and were generated by fusing 143B(TK^−^) osteosarcoma cells with enucleated patient-derived fibroblast cells harboring a 7.5-kb partial deletion in the mtDNA^74^.

For OXPHOS experiments, in addition to standard high-glucose DMEM, cells were maintained in low-glucose or galactose medium. For low-glucose medium, DMEM 1 g l^−1^ glucose (Gibco, 31885-023) was supplemented with 4.5 g l^−1^ glucose, 10% FBS and 50 μg ml^−1^ uridine. For galactose medium, no-glucose DMEM (Gibco, 11966-025) was supplemented with 10 mM d-galactose (Sigma, G0750), 110 mg l^−1^ sodium pyruvate (Gibco, 11360-070), 10% dialyzed FBS (Gibco, 26400-044) and 50 μg ml^−1^ uridine^99^.

Clonal cell populations were obtained by sorting single cells into individual wells of a flat-bottom 96-well culture plate, containing 200 μl of culture medium, using a BD FACS Melody cell sorter as described below. Then, 1 week after sorting, wells were checked for the presence of a single colony of cells to reduce the risk of obtaining nonclonal populations. Clones were expanded to 24-well plates after 2 weeks, after which further characterization (for example, heteroplasmy measurement) was performed to select suitable experimental clones.

### Plasmids and gRNA library construction

We modified CROPseq-guide-Puro (Addgene, plasmid 86708) lentiviral plasmid^22^ to embed the gRNA sequences in the 3′ UTR of a polyadenylated RFP transcript, generating the CROPseq–RFP lentiviral vector. This enables simultaneous enrichment of RFP-expressing transduced cells by fluorescence-activated cell sorting and detection of gRNA sequences through 3′ gene expression profiling. A modified RFP cDNA was a gift from F. Merkle but carried a stretch of seven adenosines, leading to spurious annealing of oligo-dT primers within the coding sequence. For future experiments, we recommend using the CROPseq–mCherry plasmid that we cloned subsequently and can be requested by contacting the corresponding author.

gRNA sequences for CROPseq (Supplementary Table 1) were designed as described previously^22^, ordered from Twist Biosciences as a ssDNA oligo pool and PCR amplified before cloning. Then, 200 fmol of the amplified oligo pool was cloned using the ClonExpress Ultra one-step cloning kit (Vazyme, C115) into 10 fmol of gel-purified CROPseq–mCherry plasmid digested by BsmBI overnight. The cloning mixture was desalted by dialyzing against nuclease-free water using a 0.05-µm membrane filter (Merck, VMWP02500) for 30 min. The desalted cloned plasmids were transformed into Endura electrocompetent cells (Lucigen, 60242-1) by electroporation in prechilled 1-mm cuvettes at 25 μF, 200 Ω, 1.5 kV before resuspending in 1 ml of recovery medium to recover and then plating or by heat shock in high-efficiency stable competent *Escherichia* *coli* (New England Biolabs (NEB), C3040H). Bacterial colonies (~43,000, estimated ~700× per gRNA) were scraped from plates. Plasmid DNA was extracted using a plasmid midi kit (Qiagen, 12143) and run on agarose gel; then, nonrecombined plasmid was extracted using a Monarch DNA gel extraction kit (NEB, T1020).

The single gRNA sequence targeting human *TFAM*^100^ was ordered as desalted oligos (Sigma) with overhangs for cloning, annealed by heating at 95 °C for 2 min and cooled at 1 °C min^−1^ to room temperature. The annealed oligo was cloned into the LentiCRISPRv2 backbone^96^ and transformed in 10-beta competent *E*. *coli* (NEB, C3019H); then, plasmid DNA was extracted using the QIAprep spin miniprep kit (Qiagen, 27104).

Single gRNA sequences targeting mouse *Tfam* and *Opa1*, as well as NT gRNA, were ordered as desalted oligos (Sigma), annealed by heating at 95 °C for 2 min and cooled at 1 °C min^−1^ to room temperature. Annealed gRNAs were cloned into LentiCRISPRv2-RFP670 (Addgene, plasmid 187646) at a 1:5 vector-to-insert ratio by Gibson assembly using the ClonExpress Ultra one-step cloning kit and transformed in 10-beta competent *E*. *coli*; then, plasmid DNA was extracted using the QIAprep spin miniprep kit (Qiagen, 27104).

The pCAG-mCherry-P2A-i4Dam and pCAG-mCherry-P2A-i4Dam-Atf4 plasmids were constructed by Gibson assembly into pCAG-IRES-GFP, using mCherry as an upstream open reading frame and Dam (Addgene, plasmid 59217)^65^ with a C-terminal Myc tag. The *Atf4* gene was amplified from mouse cDNA and cloned in frame into the pCAG-mCherry-P2A-DamID construct 3′ of Dam and the MYC tag. A P2A ribosome-skipping sequence was placed between mCherry and Dam, resulting in overexpression of Dam and ATF4 (that is, not targeted^64^), to override post-transcriptional or post-translational regulation of ATF4. To allow transient transfection of DamID^87^, an intron was inserted into Dam by ligating oligos with a modified synthetic intron (IVS)^101^ into the BamHI restriction site within Dam, between the third and fourth helices of the DNA-binding domain of the Dam methylase^102^; then, plasmids were transformed in *dam*^−^/*dcm*^−^ competent *E*. *coli* (NEB, C2925H).

### Flow cytometry

Expression of fluorescent markers was assessed using a BD LSRFortessa cell analyzer with appropriate laser, filter and detector settings. Postacquisition data analysis was performed in FlowJo version 10. Single-cell sorting was performed using a BD FACS Melody cell sorter according to the manufacturer’s instructions. In brief, samples were initially gated using doublet discrimination to select live single cells on the basis of forward scatter and side scatter parameters. If cells were being sorted on the basis of expression of a fluorescent marker, additional gating was performed to identify marker-positive cells. Cells in the desired gate were then sorted into tubes (bulk sorts) or plates (single-cell sorts), all sorts were performed in ‘single-cell’ sort mode.

### Lentiviral production and transduction

First, 24 h before transfection, HEK293T Lenti-X cells (Takara, 632180) were plated at 0.75 × 10^6^ cells per well in six-well plates. Transfections were performed with cells at 80–90% confluency using TransIT-293 transfection reagent (Mirus, MIR2704) according to the manufacturer’s instructions. The envelope plasmid (pMD2.G; Addgene, plasmid 12259), vector plasmid and packaging plasmid (psPAX2; Addgene, plasmid 12260) were added to the transfection mix in a 2:3:4 molar ratio. Supernatant containing lentiviral particles was harvested at 48 h after transfection and passed through a 40-μm filter to remove cellular debris. Isolated virus was stored at −70 °C before use in transductions. Cells to be transduced were plated at 5 × 10^4^ cells per well in 24-well plates, an appropriate volume of thawed and prewarmed lentiviral supernatant was added and the total volume was brought to 500 μl per well with fresh culture medium. Then, 3 h after transduction, an additional 500 μl of culture medium was added to bring the total volume to 1 ml. Cells were passaged to six-well plates at 24 h after transduction. To titrate lentiviral stocks, cells were transduced with increasing volumes of viral supernatant as described above. At 7 days, transduced cells were analyzed to assess expression of the transfer plasmid fluorescent marker, which acted as an indication of the transduction efficiency; this was then used to calculate the volume of viral supernatant required to achieve a given proportion of transduced cells.

### Transient transfection and DamID-seq

All transient transfections were performed using Lipofectamine 2000 (Thermo, 11668027). The m.5024C>T MEFs were plated at 2 × 10^6^ cells per well in six-well plates in 2 ml of serum-free medium immediately before transfection. Following the manufacturer’s protocol, 1 μg of each plasmid (pCAG-mCherry-p2A-i4Dam-Atf4 or pCAG-mCherry-p2A-i4Dam) prepared in *dam*^−^/*dcm*^−^ competent *E*. *coli* (NEB, C2925H) was added to each well of a six-well plate. After 5 h, the medium was replaced with DMEM plus FBS. Then, 48 h after transfection, cells were harvested and the DNA was extracted using a QiaAmp DNA micro kit (Qiagen, 56304) and processed for DamID as described previously^103^. DamID fragments were prepared for Illumina sequencing according to a modified TruSeq protocol. Sequencing was performed as paired-end 50-bp reads by the Cancer Research UK (CRUK) Genomics Core Sequencing facility on a NovaSeq 6000.

### Whole-cell 10X Genomics multiome and sequencing

Cells were prepared for 10X Genomics multiome ATAC and gene expression sequencing following a modified version of the standard 10X Genomics nuclei isolation protocol^23^. In brief, 5 × 10^5^ cells were fixed in 0.1% formaldehyde (Thermo) for 5 min at room temperature, followed by permeabilization in 0.1% NP40 (Thermo) for 3 min at 4 °C in the presence of RNase inhibitor (Roche) to prevent mRNA degradation. Fixed and permeabilized cells were resuspended in diluted nucleus buffer (10X Genomics), counted and adjusted to a concentration of 3,000 cells per μl. Following fixing and permeabilization of cells, subsequent steps were performed at the CRUK Cambridge Institute Genomics Core. Transposition, probe hybridization and ATAC and gene expression library preparation were performed using the Chromium Next GEM single-cell multiome ATAC and gene expression reagent kit (10X Genomics, PN-1000283) according to the manufacturer’s instructions (protocol no. CG000810 Rev A). Illumina sequencing was performed by the CRUK Genomics Core Sequencing facility on a NovaSeq 6000, with each run on a single lane of an SP flow cell, returning approximately 650–800 million paired-end reads per lane. Sequencing parameters were set as follows: gene expression, read 1, 28 bp; read 2, 90 bp; i5 index, 10 bp; i7 index, 10 bp; ATAC, read 1, 50 bp; read 2, 50 bp; i5 index, 16 bp; i7 index, 8 bp.

### gRNA enrichment and sequencing

CROPseq gRNA sequences were enriched from 10X Genomics multiome gene expression cDNA libraries using TAP-seq^42^. Briefly, 15 μl of cDNA from the 10X GEM reverse transcription reaction was input to a PCR reaction (TAP PCR 1) containing a CROPseq–RFP specific forward primer binding 54 bp upstream of the 3′ gRNA sequence (CROPouter) and a reverse primer binding the Illumina Truseq read 1 (partial read 1), producing an amplicon approximately 550–600 bp long covering the gRNA sequence and retaining the 3′ 10X cell barcode and unique molecular identifier sequences. To increase specificity, a second nested PCR (TAP PCR 2) was performed on the 10-ng TAP PCR 1 product using a second CROPseq–RFP-specific forward primer carrying the Illumina Truseq read 2 sequence and binding immediately upstream of the CROPseq–RFP gRNA sequence (CROPinner) and the partial read 1 reverse primer. Next, 10 ng of TAP PCR 2 product was input to a third PCR reaction (TAP PCR 3) using a forward primer carrying the Illumina P7 sequencing adaptor and a 10-bp index sequence binding the Truseq read 2 sequence (Illumina P7) and a reverse primer carrying the Illumina P5 sequencing adaptor and binding the Truseq read 1 sequence (targeted 10X). Following TAP PCR 3, enriched libraries were quantified using the KAPA library quantification kit (Roche). Sequencing on the Illumina MiSeq system was performed using the MiSeq reagent nano kit v2 for 300 cycles according to the manufacturer’s instructions. Following quantification, sequencing libraries were normalized to 4 nM, denatured, diluted to a final concentration of 10 pM and pooled with 1% 12.5 pM denatured PhiX control before sequencing. Sequencing parameters were identical to the Multiome gene expression libraries described above.

### mtDNA enrichment and sequencing

mtDNA sequences were enriched from multiome ATAC sequencing libraries by hybridization capture, using a custom xGEN hybrid capture panel (Integrated DNA Technologies) containing 270 probes targeting the mouse mtDNA sequence (average of one probe every 60 bp) (Supplementary Table 2). Hybridization capture was performed on 500 ng of ATAC sequencing library using the xGen hybridization and wash kit (Integrated DNA Technologies, 10010351) according to the manufacturer’s instructions, including the optional AMPure XP bead (Beckman Coulter, A63880) DNA concentration protocol steps. Postcapture PCR amplification was performed for 12 cycles and the final libraries were quantified using the KAPA library quantification kit and pooled for Illumina sequencing with parameters identical to the Multiome ATAC libraries described above.

### Bulk RNA-seq of single-gRNA CRISPR cells

Following lentiviral transduction with CROPseq–RFP single-gRNA CRISPR vectors, 50,000 RFP-positive cells per sample were sorted at day 6 after transduction. Total RNA was extracted from the cells using the Quick-RNA microprep kit (Zymo Research, R1050) and RNA concentration and RNA integration number equivalent (RIN^e^) values were assessed using the high-sensitivity RNA ScreenTape system (Agilent, 5067-5579/5580). RIN^e^ > 8.5 was confirmed for all samples and samples were adjusted to a final concentration of 10 ng μl^−1^ before library preparation using the NEBNext single-cell, low-input RNA library prep kit (NEB, E6420S) according to the manufacturer’s instructions. Libraries were pooled and sequenced on the Illumina NovaSeq X platform on a single lane of a 1.5B flow cell using 50-bp paired-end reads, yielding approximately 750 million reads.

### mtDNA ddPCR and pyrosequencing

Single-cell mtDNA CN measurements were made using the Bio-Rad QX200 AutoDG ddPCR system^81^. In brief, single-sorted cells in 96-well plates were lysed in lysis buffer containing 1% Tween 20 (Life Technologies, 003005) and 200 μg ml^−1^ proteinase K (Ambion, AM2546) at 37 °C for 30 min followed by 85 °C for 15 min to inactivate proteinase K. Cell lysate was then input to ddPCR reactions containing primer and probe combinations targeting the *mt-Nd1* and *mt-Co3* genes of the mouse mtDNA. Following data acquisition, the CNs obtained from the two independent mtDNA probes were averaged to give a final absolute mtDNA CN measurement for each cell.

Single-cell pyrosequencing was performed^104^ using the PyroMark Q48 Autoprep pyrosequencing system (Qiagen) according to the manufacturer’s instructions using a primer set specifically targeting the m.5024C>T mutation site.

### Long-range PCR and long-read sequencing

Genomic DNA was extracted from ear clips biopsies, taken from three m.5024C>T mice at 2 weeks of age, using the Monarch genomic DNA purification kit (NEB, T3010) and quantified by a Qubit fluorometer. A segment of mtDNA spanning positions 13200 to 5500 was amplified from 10 ng of genomic DNA by long-range PCR using PrimeSTAR GXL premix (Takara, R051B). The amplicons were isolated by Ampure XP bead cleanup and sequenced by long-read Nanopore sequencing (Plasmidsaurus, Oxford Nanopore Technologies). Reads were aligned to the GRCm39 mouse mtDNA reference genome excluding reads shorter than 8 kb. Variant positions 13614, 13715, 1781, 1866, 3009, 3823 and 5024 were extracted and converted into a binary matrix with the mutant or WT allele. For each pairwise combination of these positions, the percentage of positions matching either the WT or mutant haplotype was calculated to find the co-occurrence of alleles belonging to each haplotype.

### Western blotting and antibodies

Cultured cells were washed, dissociated using trypsin (Gibco, 15400-054) and pelleted before being snap-frozen in liquid nitrogen. Protein extraction was performed by mixing 500 μl of PathScan Sandwich ELISA lysis buffer (1×) (Cell Signaling, 7081) with each cell pellet. Following a 2-min incubation on ice, samples were spun down for 1 min at 14,000*g* at 4 °C to remove cell debris. Protein concentration was measured using the Pierce BCA protein assay kit (Thermo, 23227). NuPAGE gels were run at 165 V for 45 min. Membrane transfer was performed using an iBlot machine (Life Technologies). A 1-h incubation at room temperature in 5% milk in Tris-buffered saline with 0.1% Tween 20 was used for blocking. The membrane was incubated with the primary antibody overnight at 4 °C, while the secondary incubation was performed at room temperature for 1 h. Primary antibodies were anti-TFAM (Cell Signaling, 8076S) and anti-vinculin (Sigma, V4505). The Clean-Blot immunoprecipitate detection kit (horseradish peroxidase) (Thermo, 21232) was used for detection and imaging was conducted using the Amersham Imager 600 (General Electric).

### High-resolution respirometry

High-resolution respirometry was carried out using the O2k-Respirometer (Oroboros Instruments). Calibration was performed with DMEM plus 10% FBS. A total of 5 million cells suspended in 2 ml of medium were added to each chamber. Once the oxygen consumption rate reached a plateau, three drugs were added sequentially. First, 5 μl of oligomycin A (Merck, O4876). After establishing the new baseline, 2 μl of carbonyl cyanide *m*-chlorophenyl hydrazone (CCCP) (Merck, C2759) was added, followed up by successive doses of 1 μl until a plateau of the maximum respiratory capacity was reached. Lastly, 1 μl of rotenone (Merck, R8875) and 2 μl of antimycin A (Merck, A8674) were added in quick succession. Baseline respiration was calculated by subtracting the background (respiration still present after the addition of rotenone and antimycin A from the basal respiration of the sample, before the addition of any drugs). Proton leak was measured by subtracting the background from the respiration still present when oligomycin A was added. ATP-linked respiration was estimated by subtracting the proton leak from the basal respiration. The maximal respiratory capacity of the sample was calculated when the background was subtracted from respiration in the presence of CCCP.

### Bioinformatic analysis

#### Initial analysis and QC

Raw FASTQ files from Multiome sequencing were combined with the ‘lost reads’ FASTQ files to recover missing or low-quality reads that were initially discarded during sequencing. The FASTQ files were first run through FastQC (version 0.11.9; https://www.bioinformatics.babraham.ac.uk/projects/fastqc/) to perform QC checks on raw sequencing data and through FastQ Screen (version 0.14.1; https://www.bioinformatics.babraham.ac.uk/projects/fastq_screen/) to check for contamination. The combined FASTQ files were then processed using Cell Ranger ARC (version 2.0.1) from 10X Genomics, which performs alignment, filtering, barcode counting and peak calling, to generate feature–barcode matrices for downstream analyses. Cell Ranger ARC was first run with the standard *Mus* *musculus* reference genome (GRCm38/mm10, GENCODE vM23/Ensembl 98)^105^, before running with a custom-built *M*. *musculus* reference genome created with cellranger mkref. Some mtDNA regions exhibit low coverage because of homology with nuclear DNA, and hard-masking these NUMTs is recommended^17^. A custom blacklist (https://github.com/caleblareau/mitoblacklist/) was incorporated into the original reference, generating a hard-masked, modified *M*. *musculus* genome. Using the gex_possorted_bam.bam file, generated from the Cell Ranger ARC pipeline, the data were run through Qualimap (version 2.2.1)^106^ to evaluate the overall mapping quality of our sequencing data.

The filtered feature–barcode matrices, stored as sparse matrices in hdf5 format, were loaded into R (version 4.3.3) to perform data QC and standard preprocessing steps with the Seurat R package (version 5.1.0)^71^ for RNA data processing and the Signac R package (version 1.13.0)^107^ for ATAC data processing. Data QC was performed for both scRNA-seq and scATAC-seq data. For RNA, this involved removing cells with total RNA reads less than 1,000 and greater than 60,000, number of detected genes less than 1,000 and greater than 10,000, mitochondrial RNA percentage greater than 10 and ribosomal percentage greater than 30. For ATAC, this involved removing total ATAC reads less than 1,000 and greater than 150,000, number of accessible regions less than 500 and greater than 55,000, nucleosome banding pattern signal greater than 2 and TSS enrichment score less than 1.

Following QC, RNA-seq and ATAC-seq data were independently normalized and scaled, after which linear dimensional reduction was performed. For the RNA-seq data, cell-cycle scoring was conducted to minimize the impact of cell-cycle heterogeneity, assigning each cell a score on the basis of the expression of G2/M and S phase markers. This was initially calculated with the Seurat R package (CellCycleScoring), followed by pseudotime analysis with the tricycle R package (version 1.12.0)^72^, as described below.

As with QC, filtering and initial data processing, integration was performed separately for RNA-seq and ATAC-seq data before being combined into one multiome object for downstream analyses. RNA-seq data integration was performed by first merging the two Seurat objects, followed by SCTransform normalization while regressing out cell-cycle effects. Next, linear dimensional reduction was applied and cell clustering was determined by computing *k*-nearest neighbors and constructing a shared nearest neighbor graph. Lastly, UMAP was used for dimensionality reduction and visualization. Data integration was performed using the ‘IntegrateLayers’ function with the canonical correlation analysis (CCA) method. CCA was selected because the datasets shared common cell types while potentially containing technical or batch-related differences. This method is particularly effective for correcting subtle batch effects while preserving strong biological signals. Following integration, linear dimensional reduction, clustering and UMAP visualization were repeated. ATAC-seq data integration began by merging the two Seurat objects, followed by linear dimensional reduction, clustering and UMAP visualization. Integration was then performed using ‘FindIntegrationAnchors’ and ‘IntegrateEmbeddings’, after which linear dimensional reduction, clustering and UMAP visualization were repeated. The two integrated Seurat objects were combined and the ‘FindMultiModalNeighbours’ function was used to construct a WNN graph, followed by UMAP visualization.

For mtDNA CN analysis, mtDNA coverage is correlated with total (nuclear + mtDNA) ATAC-seq coverage per cell and others have previously included a normalization step^46^. We chose not to include normalization as it would have prevented the cell-cycle analysis in Fig. 5 and Extended Data Figs. 9 and 10 because total per-cell ATAC-seq read count increases from G1 to S and G2, in line with nuclear DNA replication (Extended Data Fig.10a,b). Normalization of the mtDNA read depth to total depth would in this case remove crucial biological variation and insight. Normalization could be applied for comparison between perturbation groups (Fig. 2j) but we opted for a homogeneous approach to estimate mtDNA CN across the manuscript.

#### gRNA detection

gRNA sequences were present in the 90-bp read 2 sequences of the gene expression library, these reads were extracted and assigned to individual cells using a bespoke pipeline. First, raw gene expression FASTQ files were processed with UMI-tools (version 1.0.1)^108^ extract to embed the 10X cell barcode sequence contained in read 1 into the corresponding read 2 read name string. Barcoded read 2 FASTQs were then aligned using bwa-mem (version 0.7.17)^109^ to a custom reference genome containing the sequence for each of the 60 gRNAs in the CROPseq library, flanked on both sides by 85-bp anchor sequences from the surrounding CROPseq–RFP backbone, ensuring sufficient reference sequence to successfully align all 90-bp read 2 sequences that overlapped at least 5 bp of the gRNA sequence. The resulting SAM output was processed in R; aligned reads were filtered to retain those with MAPQ ≥ 30 and individual reads were assigned to single cells by cross-referencing the embedded barcode sequence in the QNAME SAM field with the corresponding barcodes allocated by Cell Ranger ARC. Cells were given a single gRNA assignment if they had ≥2 separate barcoded gRNA reads identified and, in cases where multiple gRNAs were identified, the ratio of reads from the most abundant gRNA to total gRNA reads was >2:3.

#### Heteroplasmy calling

Single-cell mtDNA variant identification was performed using the mgatk package (version 0.7.0)^17^ implemented in tenx mode, with the NUMT-masked Cell Ranger ARC atac_possorted_bam.bam output file and corresponding known cell barcode list as inputs, which includes removal of PCR duplicate reads performed at the level of individual cells to give single-cell deduplicated per-base mtDNA coverage data. High-confidence heteroplasmic variants were reported in the mgatk.variant_stats.tsv output. To call single-cell heteroplasmy values, per-base mtDNA sequencing data contained in the mgatk_signac.rds output file was first imported into the Seurat object containing the corresponding Cell Ranger ARC gene expression and ATAC assays using the ReadMGATK Seurat command. Next, base calls at the seven high-confidence heteroplasmic variants corresponding to the reference and mutant alleles were extracted from the mgatk assay and the ratio of mutant to WT alleles at each variant position was used to calculate heteroplasmy. Investigation of the correlation between the heteroplasmy calls at individual variant positions, combined with previously published data^110^, confirmed that the two principal variant positions, m.5024C>T and m.13715C>T, were linked on a single mtDNA haplotype and were inversely correlated with the remaining five variants, m.1781C>T, m.1866A>G, m.3009G>T, m.3823T>C and m.13614C>T, all linked on a second mtDNA haplotype. On the basis of this confirmed linkage among all seven variants, we were able to treat the coverage at each separate variant position as an independent observation of the same underlying heteroplasmy, thus allowing us to combine the WT and mutant base calls at all seven variant sites to increase the effective coverage and maximize the accuracy of the single-cell heteroplasmy calls.

#### Heteroplasmy modeling

In silico modeling of heteroplasmy calling on the basis of subsampling of a simulated heteroplasmic mtDNA population indicated that the sample size, corresponding to the read depth at heteroplasmic sites, has a strong influence on the accuracy of single-cell heteroplasmy estimation, with greater depth resulting in increasingly accurate calls. Previous studies suggested implementing a minimum coverage cutoff of 20 reads to confidently identify a heteroplasmic variant^17,21^. To model the effect of mtDNA coverage on the accuracy of heteroplasmy calls, we generated in silico cell populations with simulated heteroplasmy values according to the distribution of heteroplasmy in our MitoPerturb-Seq dataset. On the basis of our experimental data, we used an mtDNA CN of 1,750 (measured by ddPCR; NT gRNA cells in Extended Data Fig. 7a) and a mean heteroplasmy of 58.2% with s.d. of 11.3% (calculated using all cells in the integrated dataset with combined heteroplasmic SNV coverage ≥ 20) to generate a set of normally distributed ‘true’ heteroplasmy values. To investigate the impact of sampling heteroplasmy at different mtDNA coverage, we simulated a population of 2,000 cells and randomly subsampled each cell with sample sizes of 5, 20, 50 and 100. These samples were then used to calculate ‘sampled’ heteroplasmy values for the cell and, for each sample size, the sampled heteroplasmy was plotted against the simulated true heteroplasmy for the corresponding cell, with regression line and *R*^2^ value calculated using ggplot2 (version 3.5.1)^111^ geom_smooth(). To model the likely impact of applying a coverage threshold of 20 reads to our integrated MitoPerturb-Seq dataset, we simulated a matching population of 6,510 cells (equal to the number of cells with coverage > 0 at all seven heteroplasmic SNV sites combined) as described above and sampled these cells using the same distribution of post-mtDNA enrichment coverage that we saw in our integrated data to calculate the ‘sampled’ heteroplasmy value for each cell. Sampled heteroplasmy was then plotted against the simulated true heteroplasmy, with cells sampled at depth < 20 highlighted. Together, this allowed us to conclude that, in our case, a threshold of ≥20 reads at the combined heteroplasmic variant sites represented a good compromise, effectively eliminating the cells with the most inaccurate heteroplasmy calls whilst retaining the majority of cells for downstream analysis. We note that, in datasets with higher average per-cell mtDNA coverage (for example, from cells with high-mtDNA CN, like hepatocytes^19^), it may well be possible to increase this read-depth threshold to improve overall accuracy without significantly impacting the number of cells retained in the dataset.

#### Simulating the effect of reduced sequencing depth on heteroplasmy variance

To test whether the increased heteroplasmy variance observed in *Opa1*-KD, *Polg*-KD and *Tfam*-KD cells could be attributed solely to the reduced mtDNA depth at relevant genomic sites, we performed a computational simulation modeling the expected heteroplasmy variance from sampling heteroplasmy levels in control gRNA cells at mtDNA depths characteristic of the KD cells. For each simulation, cells from the control gRNA group were sampled without replacement to match the number of cells in each KD group. For each sampled control group cell, a mtDNA depth value was sampled with replacement from the KD group to approximate the mtDNA depth distribution. A simulated alternative allele count was estimated for each cell through binomial sampling, with the sampled mtDNA depth as the number of trials and the heteroplasmy level from the sampled control cell as the probability of picking the alternative allele. The heteroplasmy level for each cell was then calculated as the ratio of its simulated alternative allele count to its assigned sampled depth. The null distribution of simulated heteroplasmy variances was generated from 5,000 simulations performed as described above and the observed *Opa1*-KD, *Polg*-KD and *Tfam*-KD heteroplasmy variance was assessed against this distribution using a two-sided empirical *P* value, adjusted for multiple comparisons using the Bonferroni–Holm method at a significance level of α = 0.05.

#### Modeling depth-dependent heteroplasmy variance

To characterize the global relationship between read depth and heteroplasmy variance, single cells were aggregated into bins of equal sample size to ensure stable variance estimates. Cells from the population without mtDNA reduction (aggregated across all gRNAs excluding *Tfam*, *Polg* and *Opa1*) were grouped into 100 bins, while the rest were grouped into 15 bins. We modeled the expected baseline variance ($\mathrm{Variance}{\rm{\propto }}\frac{1}{\mathrm{depth}}$) using quantile regression (quantreg) on the nondepleted bins, fitting the 5th and 95th percentiles. To robustly estimate uncertainty boundaries, particularly at low read depths, we calculated 95% confidence intervals (CIs) derived from 200 bootstrap iterations of resampled NT bins.

#### Cell-cycle pseudotime analysis

In addition to performing cell-cycle annotation with Seurat, we used the tricycle R package (version 1.12.0)^72^ to predict, analyze and visualize cell-cycle states in scRNA-seq. Unlike traditional cell-cycle scoring methods that rely on discrete phase markers, tricycle estimates a continuous cell-cycle trajectory using a reference-based projection approach. By leveraging a pretrained internal reference using the Runtricycle function, tricycle first projects data onto the cell-cycle embeddings using the reference and then estimates cell-cycle position. The estimated cell-cycle positions range from 0*π* to 2*π*, with 0.5*π* being the start of the S stage, *π* being the start of the G2/M stage and 1.5*π* being the middle of the M stage. The results of this function are added as metadata to the Seurat object. To evaluate tricycle’s performance, we examined the expression of key genes relative to cell-cycle position. Cell-cycle annotations from Seurat and tricycle showed high concordance, with 82.3% of annotations aligning. Of the 17.7% that did not align, 9.7% of these were a mismatch between G1 and S annotation, probably because Seurat assigns S and G2M scores on the basis of the expression of predefined marker genes, with cells exhibiting low scores for both phases inferred to be in G1 phase. Additionally, chi-square tests for independence with Bonferroni multiple testing correction were performed for each gRNA across the different cell-cycle phases to assess association between gRNA presence and cell-cycle phase distribution.

#### Mixscape analysis

For unbiased perturbation assessment from scRNA-seq datasets, we used the Mixscape method^49^, implemented within the Seurat R package. We first calculated perturbation signatures (CalcPerturbSig), setting the number of nearest neighbors to 20; when clustering by these signatures, technical variation is removed and a specific perturbation cluster is identified. Using these signatures, the RunMixscape function assumes that each target gene is a mixture of two Gaussian distributions (KO and NP) and that NP cells have the same distribution as those expressing NT gRNAs. Each cell is assigned a posterior probability of belonging to the KO group; cells with a probability greater than 0.5 are labeled KO. For the present study, Mixscape analysis was only conducted in cells that were already confidently assigned a gRNA, without cells labeled as either unknown or containing multiple gRNAs during prior gRNA assignment. All cells labeled with a negative control gene (*Eomes*, *Neurod1*, *Olig1*, *Opn4*, *Rgr* and *Rrh*) or NT gRNA were used as the control population. Following Mixscape assignment, the PlotPerturbScore function was used to examine posterior probabilities and perturbation score distributions, comparing those assigned as KO or NP to those assigned NT. Differential expression analyses were performed and visualized with the Mixscape heatmap function to see whether KO cells exhibited reduced expression. To maximize class separability, dimensionality reduction was performed with linear discriminant analysis and visualized.

#### Differential gene expression

Before differential gene expression, the PrepSCTFindMarkers function from the Seurat R package was used to prepare SCTransform-normalized data for differential gene expression analysis when using the FindMarkers set of functions. For the present data, the FindAllMarkers differential expression analysis function in Seurat was used to identify marker genes for all clusters in the single-cell dataset. This involved comparing each gRNA group to the control group (negative control genes and NT gRNAs) to identify genes that are significantly expressed in each group. Differential expression analyses were also conducted for cells classified by the Mixscape analysis as KO, comparing each gRNA with a KO classification to the control group (negative control genes and NT gRNA). Differential gene expression results were filtered for an adjusted *P* value < 0.05 and a log_2_ fold change of 0.25. We used a log_2_ fold change threshold of 0.25 (equivalent to ±20% transcript abundance) to identify genes with a significant but relatively small change in expression, which allowed us to detect consistent subtle changes across related families of genes (Supplementary Tables 4 and 5). To highlight DEGs with more significant changes in transcription, we applied an additional log_2_ fold change threshold of 0.5 (equivalent to ±50% transcript abundance) when displaying the results of the differential gene expression analysis.

Functional enrichment analysis was performed using the clusterProfiler R package (version 4.12.1)^112,113^. GO enrichment analysis was conducted with the enrichGO function, compared to a background gene set (org.Mm.eg.db, version 3.20.0). After performing enrichGO, the enrichPlot R package pairwise_termsim function with the Wang method^114^ was applied to evaluate the semantic similarity between the enriched GO terms on the basis of the topological structure of the GO graph. Relationships between the enriched GO terms were visualized as a hierarchical tree using the treeplot function from enrichPlot.

Nuclear principal component analysis (PCA) was conducted using RNA-seq data after integration, normalization and initial filtering. A linear model was fitted to the data, with corrections applied for mtDNA coverage estimated from the ATAC-seq data. For each nuclear gene *i*, we modeled the relationship between gene expression and mtDNA CN using the following linear regression: gene expression of gene *i* = β_0_ + β_1_ × mtDNA copy number + ε, where β_0_ represents the intercept, β_1_ represents the slope coefficient for mtDNA CN and ε represents the residual error term. The residuals from each linear model, representing gene expression values adjusted for mtDNA CN, were extracted and used for the subsequent analyses. The top 2,000 variable residuals from this model were used for nuclear PCA. Additionally, nuclear PCA was performed before the correction for mtDNA coverage.

#### SCENIC

Raw scRNA-seq counts from the QC filtered and integrated dataset were used as an input to the Python (version 3.12.2) pyScenic pipeline (version 0.12.1)^115^: Gene regulatory network inference was performed using the GRNBoost2 algorithm, followed by regulon prediction with the pyscenic ctx command with --mask_dropouts set to TRUE. AUCell (version 1.28.0)^60^ was used to calculate cellular regulon enrichment scores, which were then used for downstream analysis. The resulting loom file was loaded into R using SCopeLoomR (version 0.13.0; https://github.com/aertslab/SCopeLoomR). The expression matrices, regulons and AUCell matrix were extracted using get_dgem, get_regulons and get_regulons_AUC, respectively; key column attributes and metadata were also extracted. Cells were split by gRNA and for each group of cells, the AUC matrix was extracted and the mean regulon activity across all of those cells was calculated. The resulting matrix was scaled by performing z-score scaling per regulon and regulons with missing values were removed. To examine the regulon activity scores for select genes, only regulons with a relative activity score > 0 in all three genes of interest (*Opa1*, *Polg* and *Tfam*) were retained for visualization. Heat maps were generated with ComplexHeatmap (version 2.18.0)^116^.

#### Bulk RNA-seq analysis

Overall sequencing quality of Raw FASTQ files was first checked using FastQC, confirming no major issues. Next, reads were trimmed to remove Illumina TruSeq adaptor sequences using Trimmomatic (version 0.39)^117^ and aligned to the mouse GRCm38/mm10 genome reference using RUM (version 2.0.4)^118^. Aligned BAM files were used as input to HTSeq-count (version 2.0.3)^119^, using union mode, to count reads in features. Final analysis was performed in R using the EdgeR software package (version 4.4.2)^120^. To confirm the heteroplasmy level of the m.5024C>T clone 8 cells used in this experiment, we extracted reads covering the seven heteroplasmic SNV sites from the aligned BAM files of the two NT gRNA technical replicates. Five of seven sites (m.1781C>T, m.1866A>G, m.3009G>T, m.13614C>T and m.13715C>T) had a depth > 500 reads and these were used to call heteroplasmy levels, which were then averaged to give a final heteroplasmy result for each sample. Using this method, we calculated a mean heteroplasmy level of 45.6% for NT Rep 1 and 45.4% for NT Rep 2.

#### DamID-seq analysis

Raw sequencing reads from Dam-only and Dam–ATF4 samples were processed using the damidseq_pipeline (version 1.5.3)^121^. Paired-end reads were aligned to the GRCm38/mm10 reference genome using Bowtie2 (version 2.3.2)^122^ and mapped reads were assigned to GATC fragments. Signal intensities were computed in 300-bp bins using RPM (reads per million) normalization. Each Dam–ATF4 sample was normalized against each Dam-only sample, resulting in six pairwise comparisons. Peak calling on each pairwise comparison was performed using MACS3 (version 3.0.3)^123^ with the Dam–ATF4 sample used as the treatment and the corresponding Dam-only sample used as control. Peaks were called in broad mode, with a fixed fragment size of 300 bp and no model estimation (--nomodel). The effective genome size was computed from the mm10 reference genome and significance thresholds were set at *q* < 0.05 with an mfold range of 5–50. Reproducible peaks from the pairwise comparisons were identified across the biological replicates using IDR^124^, broad peaks for each Dam–ATF4 sample were merged across Dam–ATF4 versus Dam-only comparisons and deduplicated on the basis of signal strength. IDR was computed between all pairwise combinations of the three Dam–ATF4 samples using the Python IDR package (version 2.0.3) and peaks with a reproducibility score below an IDR threshold of 0.01 were retained. A multisample intersection was then performed using BEDTools (version 2.31.0)^125^ multiinter, generating a consensus peak set that captured overlapping peaks across replicates. The resulting peaks were annotated using HOMER (version 5.1)^126^ annotatePeaks.pl and motif enrichment analysis was performed using HOMER findMotifsGenome.pl with a window size of 300 bp around the peaks to identify enriched sequence motifs within the peak regions. Statistical significance of intersections between ATF4-binding targets and DEGs was performed using the R package SuperExactTest (version 1.1.2)^127^ and visualization plots were generated using ggplot2 (ref. ^111^) and ggVennDiagram (version 1.5)^128^. GO enrichment analysis was performed on genes associated with ATF4-binding sites using the clusterProfiler R package. The enrichGO function was used to identify significantly enriched biological process terms with a *q*-value threshold of 0.05. The background gene universe was set to 17,693, the number of genes detected in the 6,551 cells that were assigned a gRNA, and relationships between enriched GO terms were visualized as a hierarchical tree plot using the treeplot function in the enrichPlot R package (version 1.28.2). Gene set enrichment analysis was conducted using clusterProfiler gseGO to assess the ranking of ATF4 target genes within biological pathways. The ATF4-binding signal on the differential expressed genes was visualized as heat maps using the plotHeatmap function from deepTools (version 3.5.6)^129^. Genomic tracks for ATF4-binding signals from DamID-seq were plotted using pyGenomeTracks (version 3.9)^130^.

#### Quantification and statistical analysis

Unless described otherwise, data visualizations were generated with inbuilt package functions from the Seurat R package, including DimPlot, FeaturePlot and VlnPlot, with ggplot2 (ref. ^111^), alongside ggplot2 extensions ggalluvial (version 0.12.5)^131^, ggpmisc (version 0.6.1), ggpubr (version 0.6.0) and ggvenn (version 0.1.10), and with ComplexHeatmap^116^, enrichplot and pheatmap (version 1.0.12). Statistical tests were conducted in R; details of the tests can be found in the accompanying figure legends. Figures in this publication were created in BioRender; van den Ameele, J. https://BioRender.com/j3ro7vq and https://BioRender.com/jpd1min (2026) under license.

### Reporting summary

Further information on research design is available in the Nature Portfolio Reporting Summary linked to this article.

## Online content

Any methods, additional references, Nature Portfolio reporting summaries, source data, extended data, supplementary information, acknowledgements, peer review information; details of author contributions and competing interests; and statements of data and code availability are available at 10.1038/s41594-026-01779-7.

## Supplementary information

Supplementary Information Supplementary Table 3.

Reporting Summary

Supplementary Table Supplementary Table 1: gRNA sequences used in this study, including gRNAs used for the single-gRNA transductions and bulk RNA-sequencing. Supplementary Table 2: Hybridization capture probes for enrichment of mouse mtDNA from ATAC-seq libraries. Supplementary Table 4: DEGs (log_2_ fold change > 0.25, adjusted *P* < 0.05) from all perturbation groups. Supplementary Table 5: Differentially expressed genes (log_2_ fold change > 0.25, adjusted *P* < 0.05) in *Tfam*-KO, *Opa1*-KO, *Polg*-KO and *Atg5*-KO cells after Mixscape analysis. Supplementary Table 6: GO for DEGs in *Tfam*-KO, *Opa1*-KO, *Polg*-KO and *Atg5*-KO cells. Supplementary Table 7: Transcription factor regulon activity scores (scaled), identified by SCENIC analysis of all perturbation groups. Supplementary Table 8: ATF4 DamID-seq consensus peak coordinates. Supplementary Table 9: ATF4-bound DamID-seq genes in heteroplasmic MEFs.

Supplementary Video Time-lapse video of PIP–FUCCI-expressing HeLa cells at 20-min intervals over 24 h (related to Fig. 4).

## Source data

Source Data Fig. 5 Statistical source data.

Source Data Extended Data Fig. 1 Statistical source data.

Source Data Extended Data Fig. 4 Statistical source data.

Source Data Extended Data Fig. 7 Statistical source data.

Source Data Extended Data Fig. 9 Statistical source data.

Source Data Extended Data Fig. 9 Uncropped blot images.

Source Data Extended Data Fig. 10 Statistical source data.

## Data Availability

Further information and requests for resources and reagents should be directed to and will be fulfilled by the lead contact, J.v.d.A. (jv361@cam.ac.uk). All sequencing data were deposited to the Gene Expression Omnibus (GEO) under accession numbers GSE297416, GSE297418 and GSE297491. Additional data are supplied in the Supplementary Information. Source data are provided with this paper.

## Code availability

Custom scripts for data analysis are hosted in a dedicated publicly accessible GitHub repository (https://github.com/JvdAlab/mitoPerturb-Seq) and from Zenodo (10.5281/zenodo.19008664) ^132^.
