# Supplementary material for: MitoPerturb-Seq identifies gene-specific single-cell responses to mitochondrial DNA depletion and heteroplasmy
Source: Nat Struct Mol Biol. 2026 Apr 1;33(4):711–23. doi: 10.1038/s41594-026-01779-7 (PMC13095666; doi:10.1038/s41594-026-01779-7)
Supplement: Supplementary file 1 — Supplementary Table 3. [file 41594_2026_1779_MOESM1_ESM.pdf]

nature structural & molecular biology

Supplementary information https://doi.org/10.1038/s41594-026-01779-7

MitoPerturb-Seq identifies gene-specific single-cell responses to mitochondrial DNA depletion and heteroplasmy

In the format provided by the authors and unedited

Supplementary Table 3: gRNA & Perturbation Group Cell Numbers

Guide Group	Cell No.	Perturbation Group	Cell No.
Akap1-1	119	Akap1	371
Akap1-3	147		
Akap1-4	105		
Nnt-2	192	Nnt	440
Nnt-5	129		
Nnt-6	119		
Polg-4	178	Polg (No Polg-6)	286
Polg-5	108		
Polg-6	116		
Tfam-1	75	Tfam	270
Tfam-2	121		
Tfam-3	74		
Dnm1l-1	114	Dnm1l	405
Dnm1l-2	151		
Dnm1l-3	140		
Mtfp1-2	156	Mtfp1	303
Mtfp1-3	56		
Mtfp1-6	91		
Opa1-1	85	Opa1	308
Opa1-3	78		
Opa1-5	145		
Snx9-2	85	Snx9	283
Snx9-3	85		
Snx9-6	113		
Atg5-3	107	Atg5	313
Atg5-4	99		
Atg5-5	107		
Oma1-2	176	Oma1	377
Oma1-3	91		
Oma1-5	110		
Pink1-2	129	Pink1	469
Pink1-4	203		
Pink1-5	137		
Ppargc1a-2	111	Ppargc1a	317
Ppargc1a-3	125		
Ppargc1a-6	81		
Prkn-3	126	Prkn	352
Prkn-4	124		
Prkn-6	102		
Eomes-1	71	Eomes	239
Eomes-2	89		
Eomes-3	79		
Neurod1-2	101	Neurod1	294
Neurod1-3	96		
Neurod1-4	97		
Olig1-2	103	Olig1	264
Olig1-3	115		
Olig1-4	46		
Opn4-2	101	Opn4	297
Opn4-3	59		
Opn4-6	137		
Rgr-1	153	Rgr	307
Rgr-2	73		
Rgr-3	81		
Rrh-1	64	Rrh	250
Rrh-3	50		
Rrh-6	136		
Nontargeting-1	95	Nontargeting	290
Nontargeting-2	56		
Nontargeting-3	139		
