# Supplementary material for: MitoPerturb-Seq identifies gene-specific single-cell responses to mitochondrial DNA depletion and heteroplasmy
Source: Nat Struct Mol Biol. 2026 Apr 1;33(4):711–23. doi: 10.1038/s41594-026-01779-7 (PMC13095666; doi:10.1038/s41594-026-01779-7)
Supplement: Supplementary file 2 — Reporting Summary [file 41594_2026_1779_MOESM2_ESM.pdf]

nature portfolio

Corresponding author(s): Patrick F Chinnery
Jelle van den Ameele

Last updated by author(s): Dec 2, 2025

Reporting Summary

Nature Portfolio wishes to improve the reproducibility of the work that we publish. This form provides structure for consistency and transparency in reporting. For further information on Nature Portfolio policies, see our Editorial Policies and the Editorial Policy Checklist.

Statistics

For all statistical analyses, confirm that the following items are present in the figure legend, table legend, main text, or Methods section.

n/a	Confirmed	
☐	☒	The exact sample size (*n*) for each experimental group/condition, given as a discrete number and unit of measurement
☐	☒	A statement on whether measurements were taken from distinct samples or whether the same sample was measured repeatedly
☐	☒	The statistical test(s) used AND whether they are one- or two-sided<br>*Only common tests should be described solely by name; describe more complex techniques in the Methods section.*
☒	☐	A description of all covariates tested
☐	☒	A description of any assumptions or corrections, such as tests of normality and adjustment for multiple comparisons
☐	☒	A full description of the statistical parameters including central tendency (e.g. means) or other basic estimates (e.g. regression coefficient) AND variation (e.g. standard deviation) or associated estimates of uncertainty (e.g. confidence intervals)
☐	☒	For null hypothesis testing, the test statistic (e.g. *F*, *t*, *r*) with confidence intervals, effect sizes, degrees of freedom and *P* value noted<br>*Give P values as exact values whenever suitable.*
☒	☐	For Bayesian analysis, information on the choice of priors and Markov chain Monte Carlo settings
☒	☐	For hierarchical and complex designs, identification of the appropriate level for tests and full reporting of outcomes
☐	☒	Estimates of effect sizes (e.g. Cohen's *d*, Pearson's *r*), indicating how they were calculated

Our web collection on statistics for biologists contains articles on many of the points above.

Software and code

Policy information about availability of computer code

Data collection	NovaSeq Control Software v1.7.5
Data analysis	All software packages used for data analysis are listed and referenced in the methods section, a list with software versions is provided below. Complete code used for bespoke data analysis are available via GitHub (https://github.com/JvdAlab/mitoPerturb-Seq).<br>R Statistical Software (v4.3.3), Python (v3.12.2), Seurat (v5.1.0), Signac (v1.13.0), pySCENIC (v0.12.1), AUCell (v1.28.0), tricycle (v1.12.0), Cell Ranger ARC (v2.0.1), mgatk (v0.7.0), UMI-Tools (v1.0.1), FastQC (v0.11.9), FastQ Screen (v0.14.1), Qualimap (v2.2.1), BWA (v0.7.17), Bowtie2 (v2.4.5), clusterProfiler (v4.12.1), enrichR (v3.2), Trimmomatic (v0.39), RUM (v2.0.4), HTSeq (v2.0.3), edgeR (v4.4.2), damidseq_pipeline (v1.5.3), MACS (v3.0.3), IDR (v2.0.3), bedtools (v2.31.0), HOMER (v5.1), SuperExactTest (v1.1.2), ggplot2 (v3.5.1), ggVennDiagram (v1.5), enrichplot (v1.10.1), deepTools (v3.5.6), pyGenomeTracks (v3.9), ggalluvial (v0.12.5), ggpmisc (v0.6.1), ggpubr (v0.6.0), ggvenn (v0.1.10), ComplexHeatmap (v2.22.0), Pheatmap (v1.0.12), quantreg (v6.1), PyroMark Analysis Software (QIAGEN), FlowJo (v10), QX Manager (Bio-Rad)

For manuscripts utilizing custom algorithms or software that are central to the research but not yet described in published literature, software must be made available to editors and reviewers. We strongly encourage code deposition in a community repository (e.g. GitHub). See the Nature Portfolio guidelines for submitting code & software for further information.

Data

Policy information about availability of data

All manuscripts must include a data availability statement. This statement should provide the following information, where applicable:

- Accession codes, unique identifiers, or web links for publicly available datasets
- A description of any restrictions on data availability
- For clinical datasets or third party data, please ensure that the statement adheres to our policy

The 10X Multiome Mitoperturb-Seq, Bulk RNAseq and DamID-seq data produced in this study have been deposited into the Gene Expression Omnibus database and are accessible via the following accession numbers:
10X Multiome ATAC & GEX (MitoPerturb-seq): GSE297418
Tfam/Opa1 CRISPR bulk RNAseq: GSE297416
Atf4 DamID-seq: GSE297491

Research involving human participants, their data, or biological material

Policy information about studies with human participants or human data. See also policy information about sex, gender (identity/presentation), and sexual orientation and race, ethnicity and racism.

Reporting on sex and gender	No reporting on sex and gender is included in the study.
Reporting on race, ethnicity, or other socially relevant groupings	No reporting on race, ethnicity, or other socially relevant groupings is included in the study.
Population characteristics	See above.
Recruitment	No recruitment was involved in this study.
Ethics oversight	No ethical approval was required for the study.

Note that full information on the approval of the study protocol must also be provided in the manuscript.

Field-specific reporting

Please select the one below that is the best fit for your research. If you are not sure, read the appropriate sections before making your selection.

☒ Life sciences ☐ Behavioural & social sciences ☐ Ecological, evolutionary & environmental sciences

For a reference copy of the document with all sections, see nature.com/documents/nr-reporting-summary-flat.pdf

Life sciences study design

All studies must disclose on these points even when the disclosure is negative.

Sample size	Mitoperturb-Seq on MEF cells yielded 11,990 single-cells with an average of 328 cells assigned to each CRISPR gene knockout group, each cell represents an individual biological replicate in this experiment.
Data exclusions	QC of single-cell RNAseq & ATACseq data was performed to remove poor-quality cells from the final dataset, standard thresholds for filtering single-cell data were employed and are described in the methods section "Initial Analysis & Quality Control".
Replication	Mitoperturb-Seq was performed in technical duplicate to yield the 11,990 single-cells described above, no significant batch effects were observed between the two replicates. Bulk RNAseq validation experiments were performed in biological duplicate (two independent transductions per guide RNA) using validated gRNA sequences from the Mitoperturb-Seq pooled library, and were performed in an independent population of MEF cells to those used for the Mitoperturb-Seq experiments, no significant batch effects were seen between the biological replicates upon analysis of the data.
Randomization	Cells in the Mitoperturb-Seq experiments were randomly allocated to gRNA groups by the nature of the CRISPR screening process, therefore we did not need to actively randomise these samples.
Blinding	Blinding was not necessary due to the random nature of the CRISPR gRNA transduction, as described above.

Reporting for specific materials, systems and methods

We require information from authors about some types of materials, experimental systems and methods used in many studies. Here, indicate whether each material, system or method listed is relevant to your study. If you are not sure if a list item applies to your research, read the appropriate section before selecting a response.

Materials & experimental systems

n/a	Involved in the study
☐	☒ Antibodies
☐	☒ Eukaryotic cell lines
☒	☐ Palaeontology and archaeology
☐	☒ Animals and other organisms
☒	☐ Clinical data
☒	☐ Dual use research of concern
☒	☐ Plants

Methods

n/a	Involved in the study
☒	☐ ChIP-seq
☐	☒ Flow cytometry
☒	☐ MRI-based neuroimaging

Antibodies

Antibodies used

Anti-TFAM (D5C8) Rabbit mAb, Cell Signaling #8076S, used at 1:1000 dilution.
Anti-Vinculin Mouse mAb, Sigma #V4505, used at 1:1000 dilution.
Anti-Mouse Ig/HRP Rabbit pAb, Agilent #P0260, used at 1:1000 dilution.
Anti-Rabbit Ig/HRP Swine pAb, Agilent #P0217, used at 1:1000 dilution.

Validation

Anti-TFAM (D5C8) Rabbit mAb has been validated by the manufacturer for use in western blot with confirmed cross-reactivity to human TFAM (https://www.cellsignal.com/products/primary-antibodies/tfam-d5c8-rabbit-mab/8076), in the study we included cell lysate from WT HeLa cells as a positive control for TFAM staining when assessing CRISPR KO efficiency.
Anti-Vinculin Mouse mAb has been validated by the manufacturer for use in western blot with confirmed cross-reactivity to human VCL (Vinculin, https://www.sigmaaldrich.com/GB/en/product/sigma/v9131)

Eukaryotic cell lines

Policy information about cell lines and Sex and Gender in Research

Cell line source(s)

m.5024C>T Mouse Embryonic Fibroblasts were isolated from a female embryo and immortalized by Dr JB Stewart, University of Newcastle
WT HeLa cells, originally from a female patient, were purchased from the European Collection of Authenticated Cell Cultures, catalogue number 93021013
HEK 293T cells, originally from a female fetus, were purchased from Takara (Lenti-X 293T Cells), catalogue number 632180
DeltaH2.1 cybrid cells were generated by Dr CT Moraes by fusing 143B(TK-) osteosarcoma cells (female) with enucleated patient-derived fibroblast cells harboring a 7.5 kb partial deletion in the mtDNA, the source of these cells is referenced in the manuscript.

Authentication

The MEF cells were authenticated by the presence of 7 specific SNPs in the mtDNA sequence, which were confirmed as part of the analytical pipeline employed in the study.
Human cell lines used in the study were purchased from verified sources:
WT HeLa cells - ECACC, #93021013
HEK 293T cells - Takara, Lenti-X 293T #632180
DeltaH2.1 cybrid cells were authenticated by the presence of the 7.5Kb partial deletion in the mtDNA, which was confirmed by ddPCR assays performed in the study.

Mycoplasma contamination

All cell lines used in this study were tested for mycoplasma and were confirmed to be negative prior to performing experiments.

Commonly misidentified lines
(See ICLAC register)

None of the cell lines used appear in version 13 of the ICLAC Register of Misidentified Cell Lines

Animals and other research organisms

Policy information about studies involving animals; ARRIVE guidelines recommended for reporting animal research, and Sex and Gender in Research

Laboratory animals

The mouse line used in this study was m.5024C>T (Allele symbol: mt-Tam1Jbst, MGI ID: 5902095), bred on the C57BL/6 background

Wild animals

The study did not involve wild animals.

Reporting on sex

No reporting on sex is included in the study.

Field-collected samples

The study did not involve samples collected in the field.

Ethics oversight

Mouse husbandry was performed in a Home Office-designated facility, according to the UK Home Office guidelines upon approval by

Ethics oversight	the University of Cambridge Animal Welfare & Ethical Review Body (AWERB) and the UK Home Office (project license P6C97520A/ PP8565009, Protocol 5 - Breeding and Maintenance of Genetically Modified Animals).

Note that full information on the approval of the study protocol must also be provided in the manuscript.

Plants

Seed stocks	Plants were not used in this study, as highlighted under Materials & experimental systems
Novel plant genotypes	Plants were not used in this study, as highlighted under Materials & experimental systems
Authentication	Plants were not used in this study, as highlighted under Materials & experimental systems

Flow Cytometry

Plots

Confirm that:

☒ The axis labels state the marker and fluorochrome used (e.g. CD4-FITC).

☒ The axis scales are clearly visible. Include numbers along axes only for bottom left plot of group (a 'group' is an analysis of identical markers).

☐ All plots are contour plots with outliers or pseudocolor plots.

☒ A numerical value for number of cells or percentage (with statistics) is provided.

Methodology

Sample preparation	For CROP-RFP expressing MEF cells, and FUCCI expressing HeLa/HEK293T/DeltaH2.1 cells, samples were prepared from in vitro cell cultures by first trypsinising cells, followed by washing 2x in PBS, followed by resuspension in 500ul PBS per sample. Finally, cells were passed through a 50um filter prior to analysis/sorting on the BD FACSMelody Cell Sorter
Instrument	BD FACSMelody Cell Sorter
Software	FlowJo (v10)
Cell population abundance	For the CROP-RFP MEF cell sorts, cells were sorted in 'single-cell' mode and selected for expression of the RFP marker carried on the gRNA lentiviral backbone, since this marker is only expressed following successful integration of the gRNA cassette, this ensured that the post-sort populations collected for MitoPerturb-seq fixation/permeabilisation were highly enriched (close to 100% pure) for single cells expressing a CRISPR gRNA.
Gating strategy	For all flow cytometry experiments, cell populations were initially gated based on 2-dimensional FSC-A/SSC-A signal intensity to select the live-cell population, further gating based on SSC-H/SSC-A was applied to select single cells and exclude doublets. For CROP-RFP MEF sorts, RFP-positive cells were gated by visualising RFP signal on a histogram and applying a sort gate based on the clear division between the RFP -ve and RFP +ve cell populations. For FUCCI HeLa cell sorts, FUCCI signal was visualised on a 2-dimensional mVenus/mCherry plot, with sort gates placed around the G1, S & G2M cell populations as outlined in the manuscript.

☒ Tick this box to confirm that a figure exemplifying the gating strategy is provided in the Supplementary Information.
