# Supplementary figures and images for: MitoPerturb-Seq identifies gene-specific single-cell responses to mitochondrial DNA depletion and heteroplasmy
Source: Nat Struct Mol Biol. 2026 Apr 1;33(4):711–23. doi: 10.1038/s41594-026-01779-7 (PMC13095666; doi:10.1038/s41594-026-01779-7)

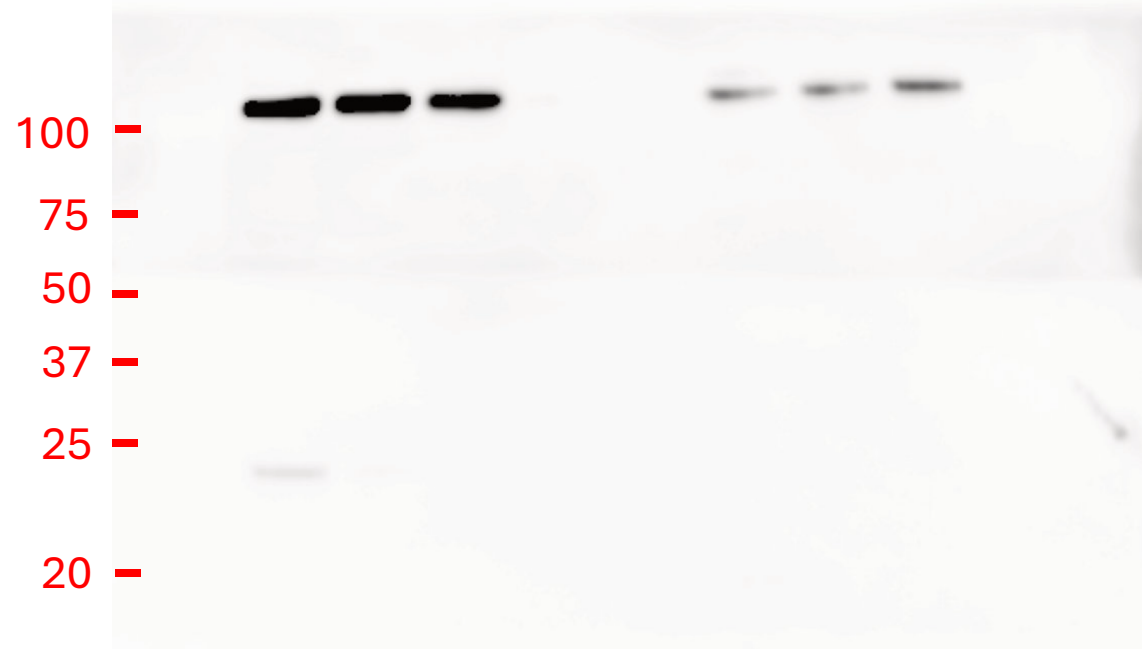

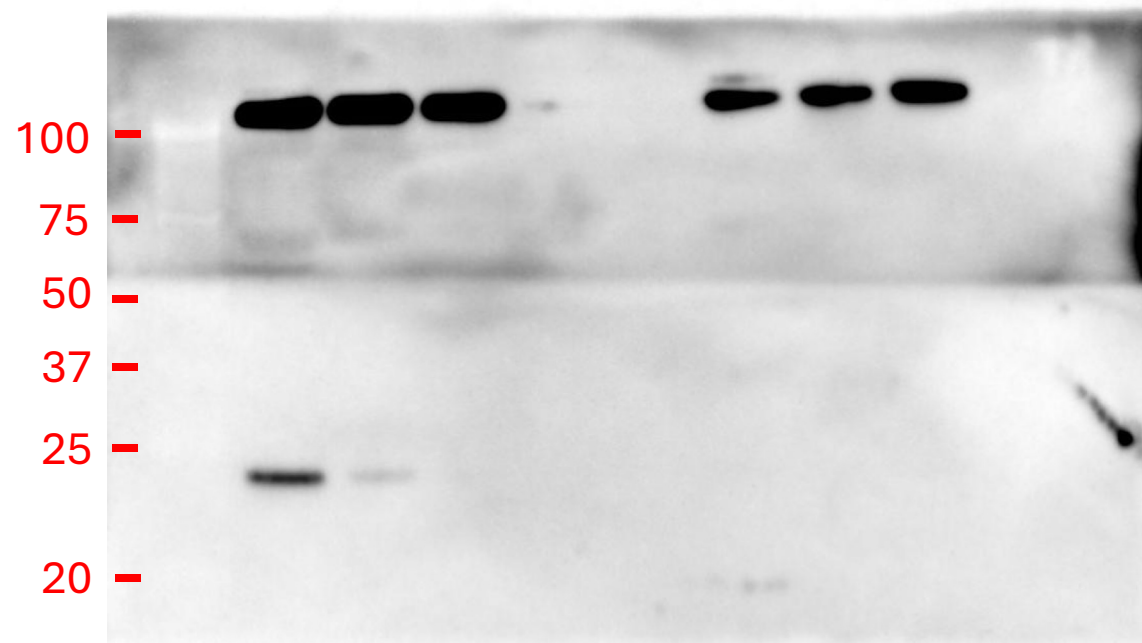

100 —  
75 —  
50 —  
37 —  
25 —  
20 —

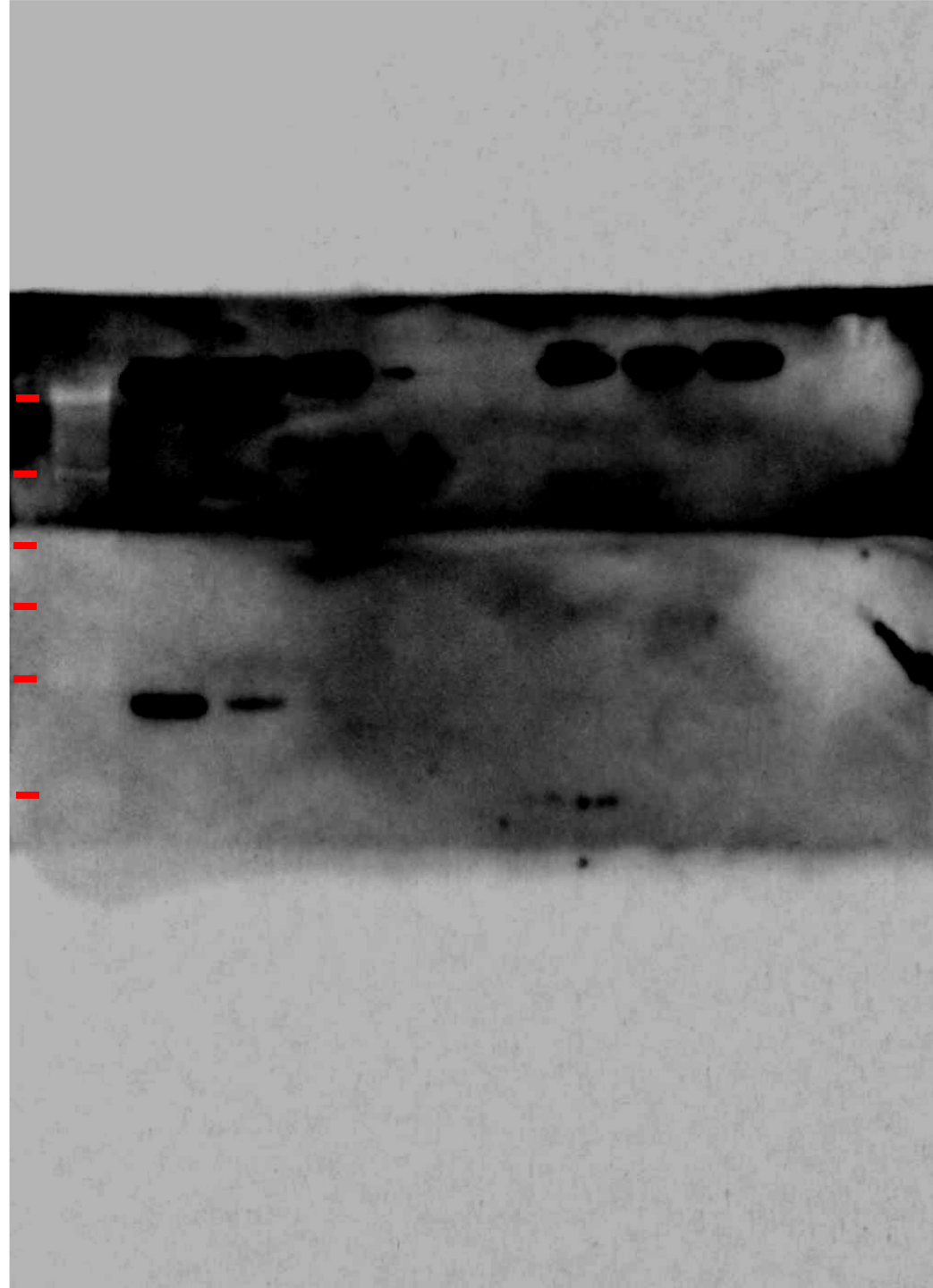

Supplement: Supplementary file 10 — Uncropped blot images. [file 41594_2026_1779_MOESM10_ESM.pdf]
